# Supplementary material for: Genome-wide association studies identify 137 genetic loci for DNA methylation biomarkers of aging
Source: Genome Biol. 2021 Jun 29;22:194. doi: 10.1186/s13059-021-02398-9 (PMC8243879; doi:10.1186/s13059-021-02398-9)
Supplement: Supplementary file 3 — Additional file 3. Supplementary Figures - Figures S1-S31. [file 13059_2021_2398_MOESM3_ESM.pdf]

Additional File 3: Supplementary Figures

Fig S1: QQ Plot for DNAm granulocyte proportions in the European ancestry GWAS meta-analysis.
Fig S2: QQ Plot for GrimAge Acceleration in the European ancestry GWAS meta-analysis.
Fig S3: QQ Plot for Hannum Age Acceleration in the European ancestry GWAS meta-analysis.
Fig S4: QQ Plot for IEAA in the European ancestry GWAS meta-analysis
Fig S5: QQ Plot for DNAm PAI1 levels in the European ancestry GWAS meta-analysis.
Fig S6: QQ Plot for PhenoAge Acceleration in the European ancestry GWAS meta-analysis.
Fig S7: QQ Plot for DNAm granulocyte proportions in the African American GWAS meta-analysis.
Fig S8: QQ Plot for GrimAge Acceleration in the African American GWAS meta-analysis.
Fig S9: QQ Plot for Hannum Age Acceleration in the African American GWAS meta-analysis.
Fig S10: QQ Plot for IEAA in the African American GWAS meta-analysis.
Fig S11: QQ Plot for PAI1 in the African American GWAS meta-analysis.
Fig S12: QQ Plot for PhenoAge Acceleration in the African American GWAS meta-analysis.
Fig S13: Mean/SD epigenetic age plotted against mean/SD chronological age in European ancestry and African American cohorts.
Fig S14: Manhattan Plot for DNAm granulocyte proportions in the European ancestry GWAS meta-analysis.
Fig S15: Manhattan Plot for GrimAge Acceleration in the European ancestry GWAS meta-analysis.
Fig S16: Manhattan Plot for Hannum Age Acceleration in the European ancestry GWAS meta-analysis.
Fig S17: Manhattan Plot for IEAA in the European ancestry GWAS meta-analysis.
Fig S18: Manhattan Plot for DNAm PAI1 levels in the European ancestry GWAS meta-analysis.
Fig S19: Manhattan Plot for PhenoAge Acceleration in the European ancestry GWAS meta-analysis.
Fig S20: Plot of effect sizes for genome-wide significant SNPs in Gibson et al. vs effect sizes in a lookup of the current meta-analysis results for Hannum Age Acceleration and IEAA.
Fig S21: Plot of effect sizes for genome-wide significant SNPs in Astle et al. vs effect sizes in a lookup of the current meta-analysis results for DNAm granulocyte proportions (and vice versa).
Fig S22: Manhattan Plot for DNAm granulocyte proportions in the African American GWAS meta-analysis.
Fig S23: Manhattan Plot for GrimAge Acceleration in the African American GWAS meta-analysis.
Fig S24: Manhattan Plot for Hannum Age Acceleration in the African American GWAS meta-analysis.
Fig S25: Manhattan Plot for IEAA in the African American GWAS meta-analysis.
Fig S26: Manhattan Plot for DNAm PAI1 levels in the African American GWAS meta-analysis.
Fig S27: Manhattan Plot for PhenoAge Acceleration in the African American GWAS meta-analysis.
Fig S28: Plot of lead African American and European ancestry trans-ethnic meta-analysis SNP effect sizes against the same SNPs in the Hispanic subset of the MESA cohort.
Fig S29: Lookup of 24 blood-based independent genome-wide significant SNPs for IEAA in a brain-based GWAS of IEAA (overlap of 21 SNPs). The red line represents the linear regression line (r=0.74) for SNPs that are also mQTLs for IEAA clock CpG sites. The turquoise line represents the linear regression line (r=0.08) for SNPs that are not mQTLs for IEAA clock CpGs. Labelled points correspond to loci where there was strong evidence of SNPs sharing genetic effects with eQTLs.
Fig S30: LocusZoom plot for the region (*DSRC6*/*RIPPLY3*) with highest evidence of genetic colocalization for the blood- and brain-based GWASs. Note that the lead SNP from the blood-based GWAS also colocalizes with a mQTL for an IEAA clock CpG, cg13450409 (PP=0.99, **Additional File 2: Table S11**).
Fig S31: LD regression SNP-based heritability estimates for the six epigenetic biomarkers.

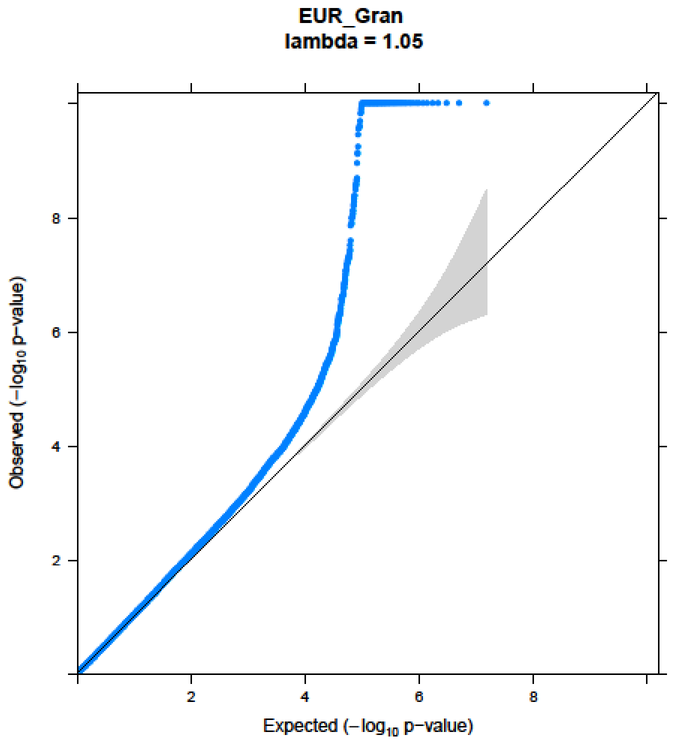

Fig S1: QQ Plot for DNAm granulocyte proportions in the European ancestry GWAS meta-analysis.

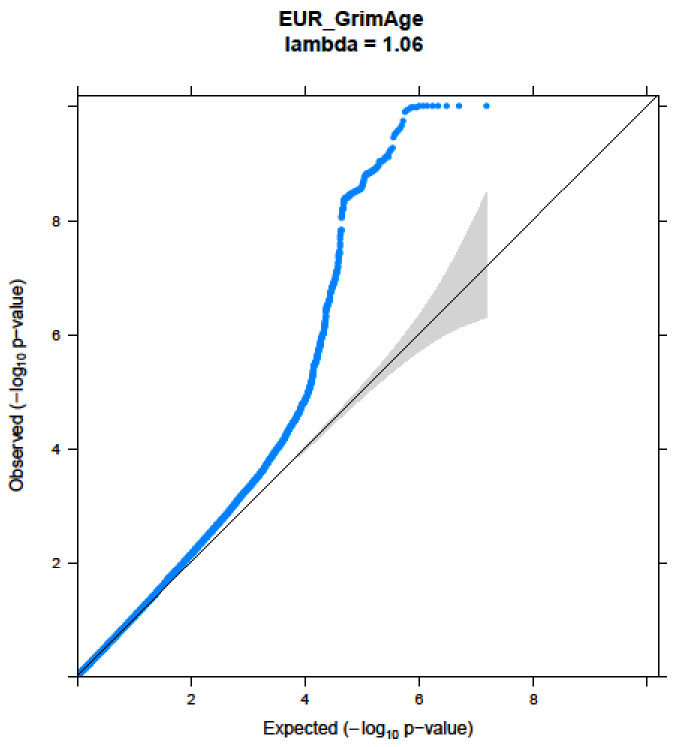

Fig S2: QQ Plot for GrimAge Acceleration in the European ancestry GWAS meta-analysis.

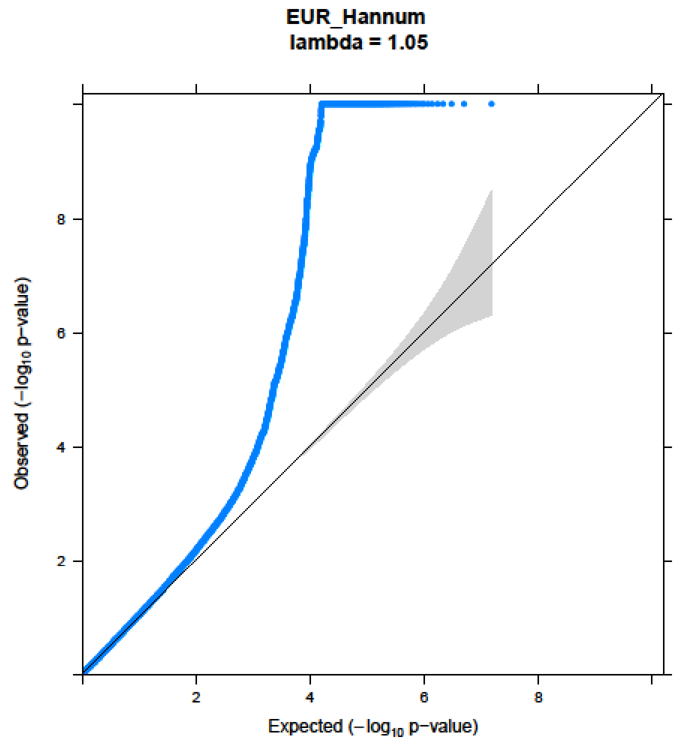

Fig S3: QQ Plot for Hannum Age Acceleration in the European ancestry GWAS meta-analysis.

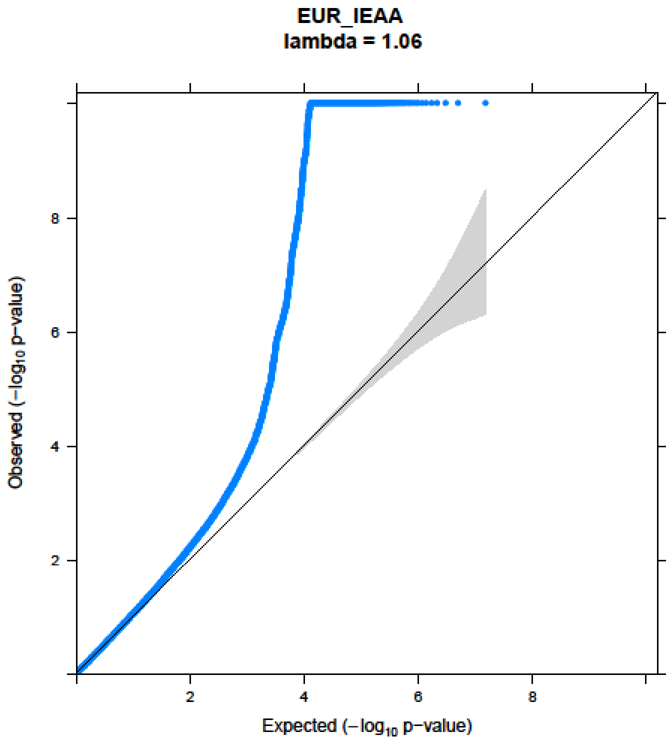

Fig S4: QQ Plot for IEAA in the European ancestry GWAS meta-analysis

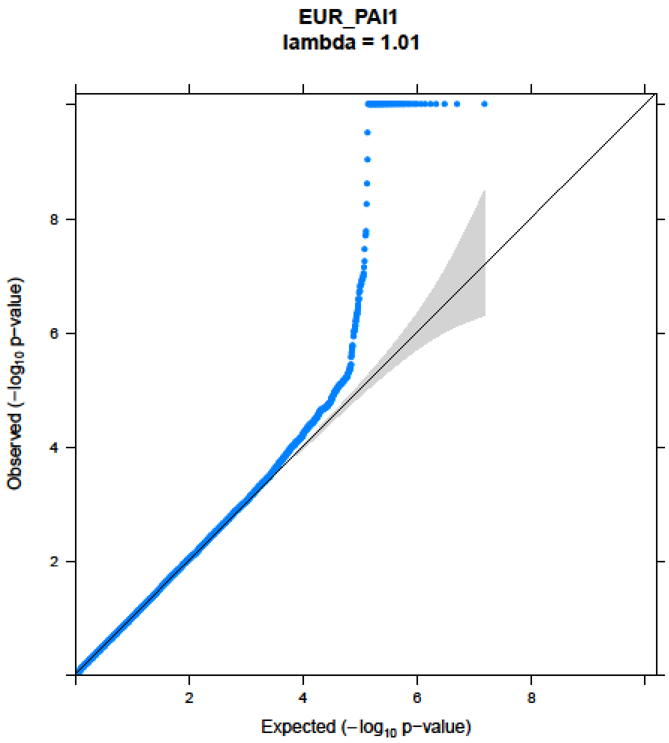

Fig S5: QQ Plot for DNAm PAI1 levels in the European ancestry GWAS meta-analysis.

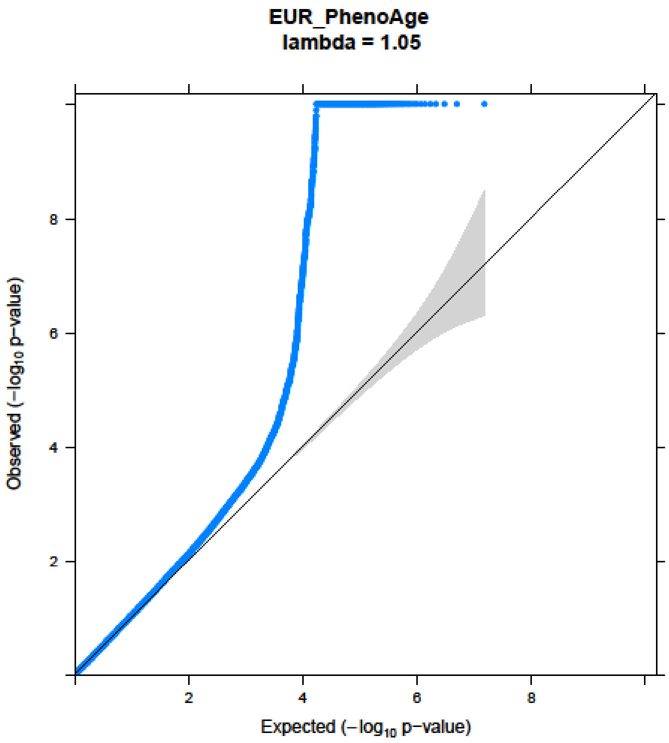

Fig S6: QQ Plot for PhenoAge Acceleration in the European ancestry GWAS meta-analysis.

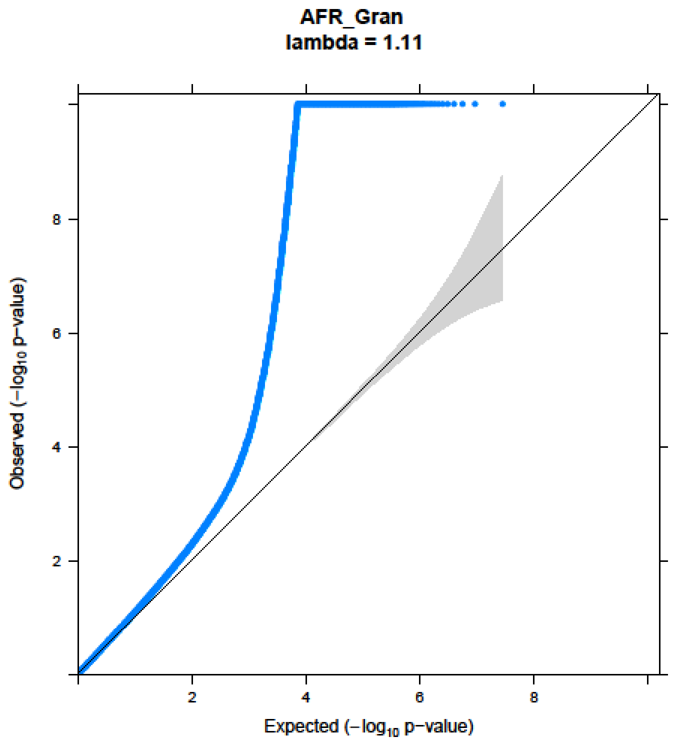

Fig S7: QQ Plot for DNAm granulocyte proportions in the African American GWAS meta-analysis.

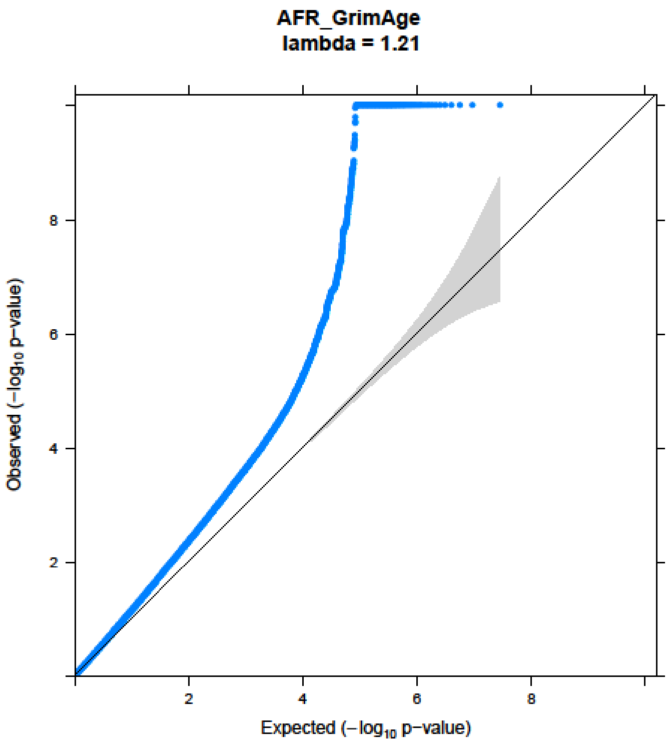

Fig S8: QQ Plot for GrimAge Acceleration in the African American GWAS meta-analysis.

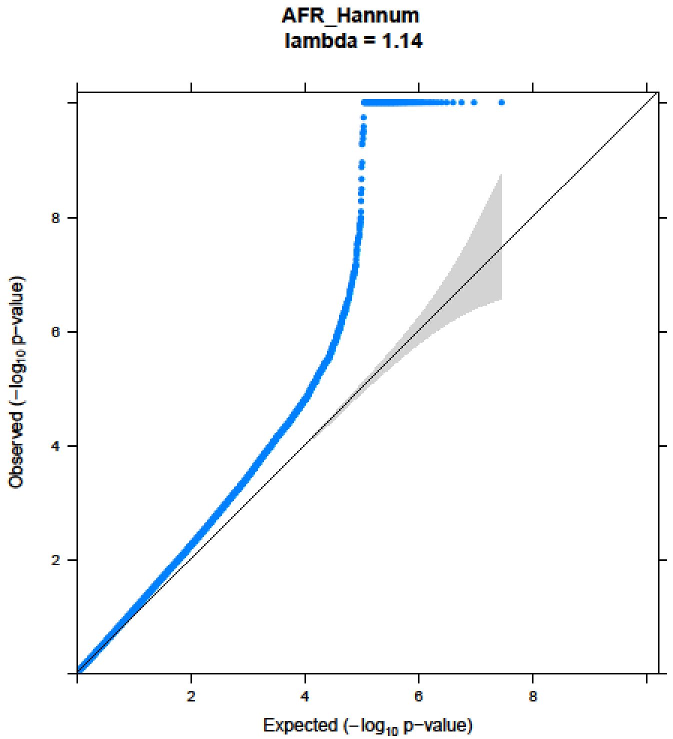

Fig S9: QQ Plot for Hannum Age Acceleration in the African American GWAS meta-analysis

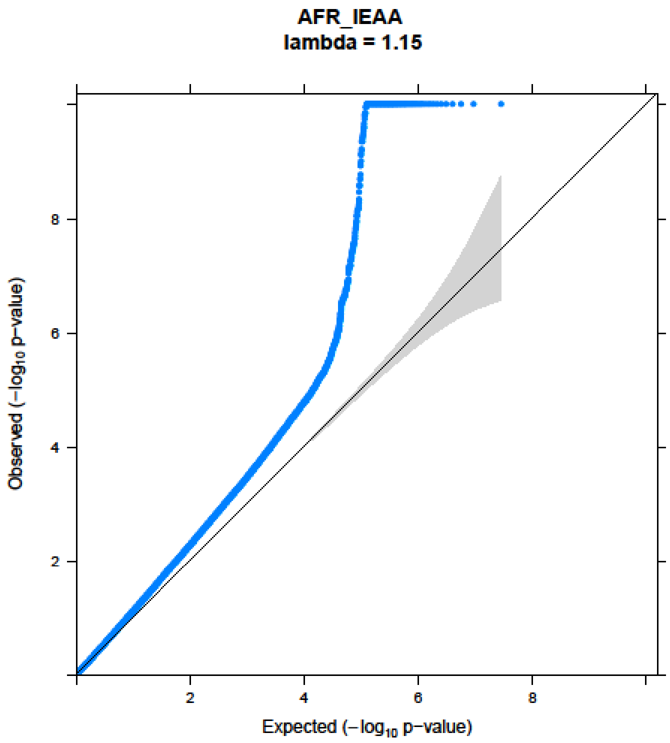

Fig S10: QQ Plot for IEAA in the African American GWAS meta-analysis.

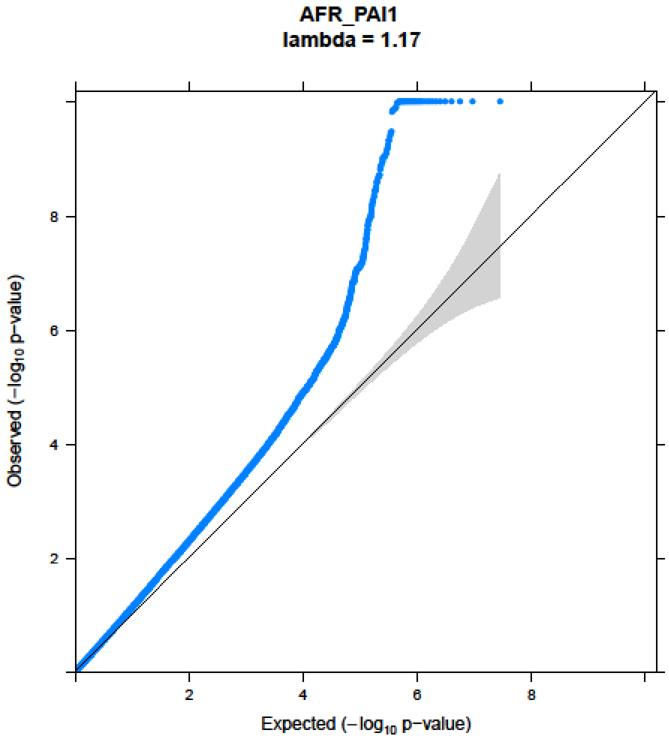

Fig S11: QQ Plot for DNAm PAI1 levels in the African American GWAS meta-analysis.

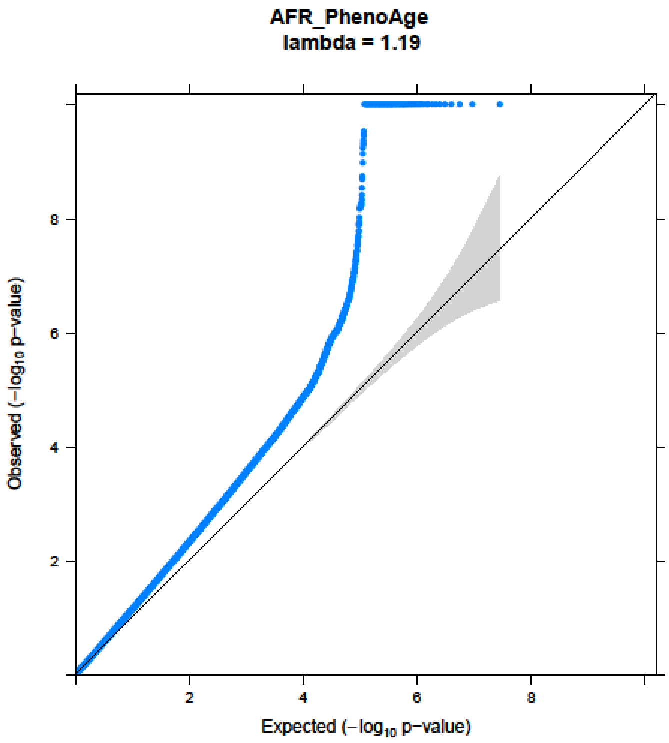

Fig S12: QQ Plot for PhenoAge Acceleration in the African American GWAS meta-analysis.

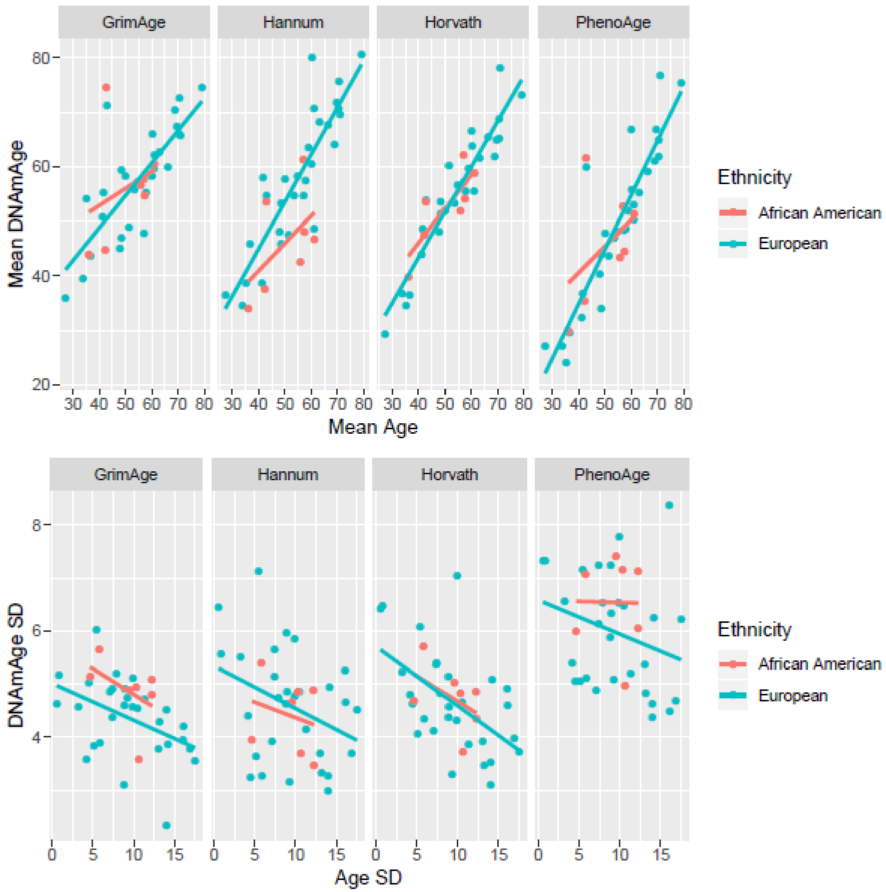

Fig S13: Mean/SD epigenetic age plotted against mean/SD chronological age in European ancestry and African American cohorts.

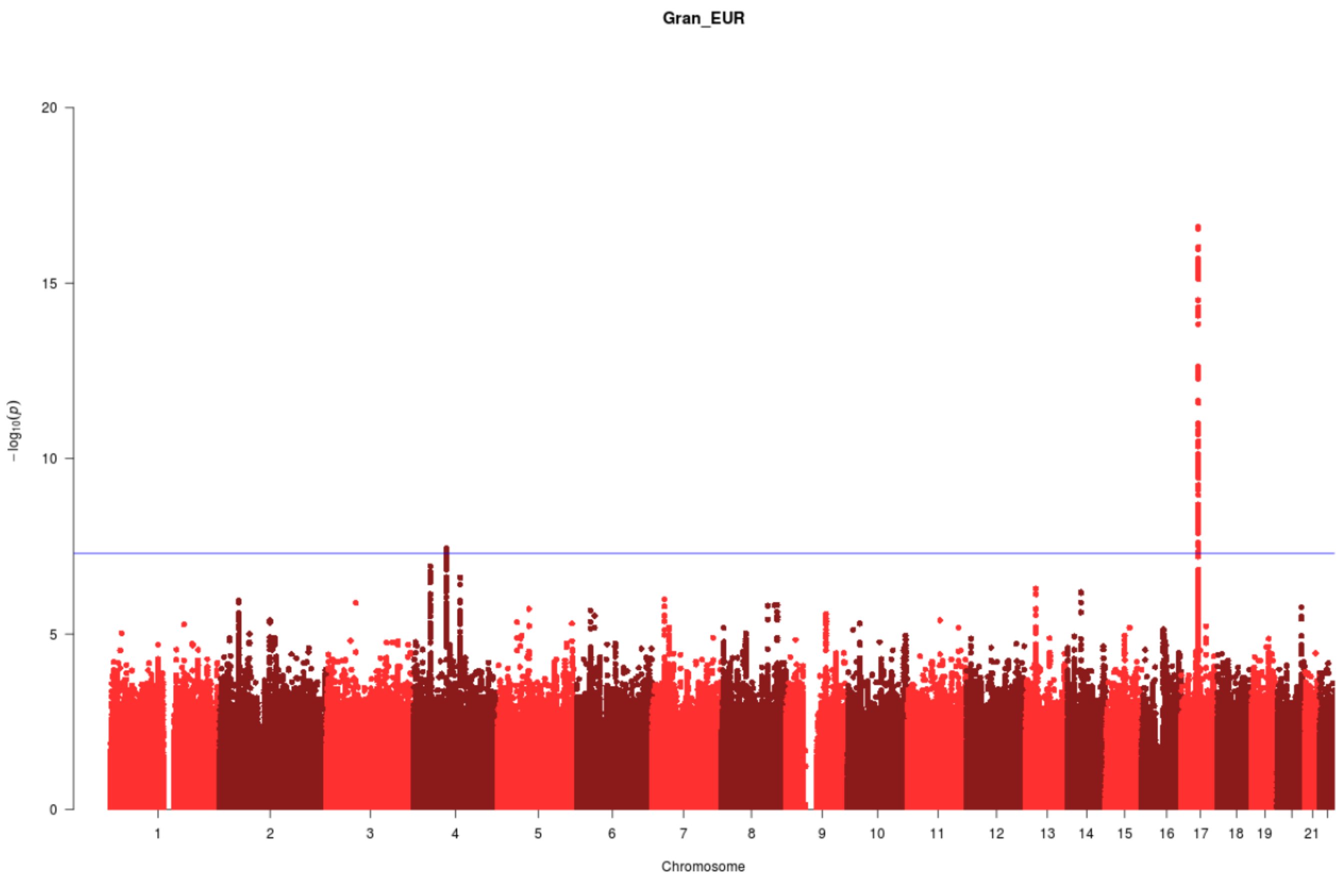

Fig S14: Manhattan Plot for DNAm granulocyte proportions in the European ancestry GWAS meta-analysis.

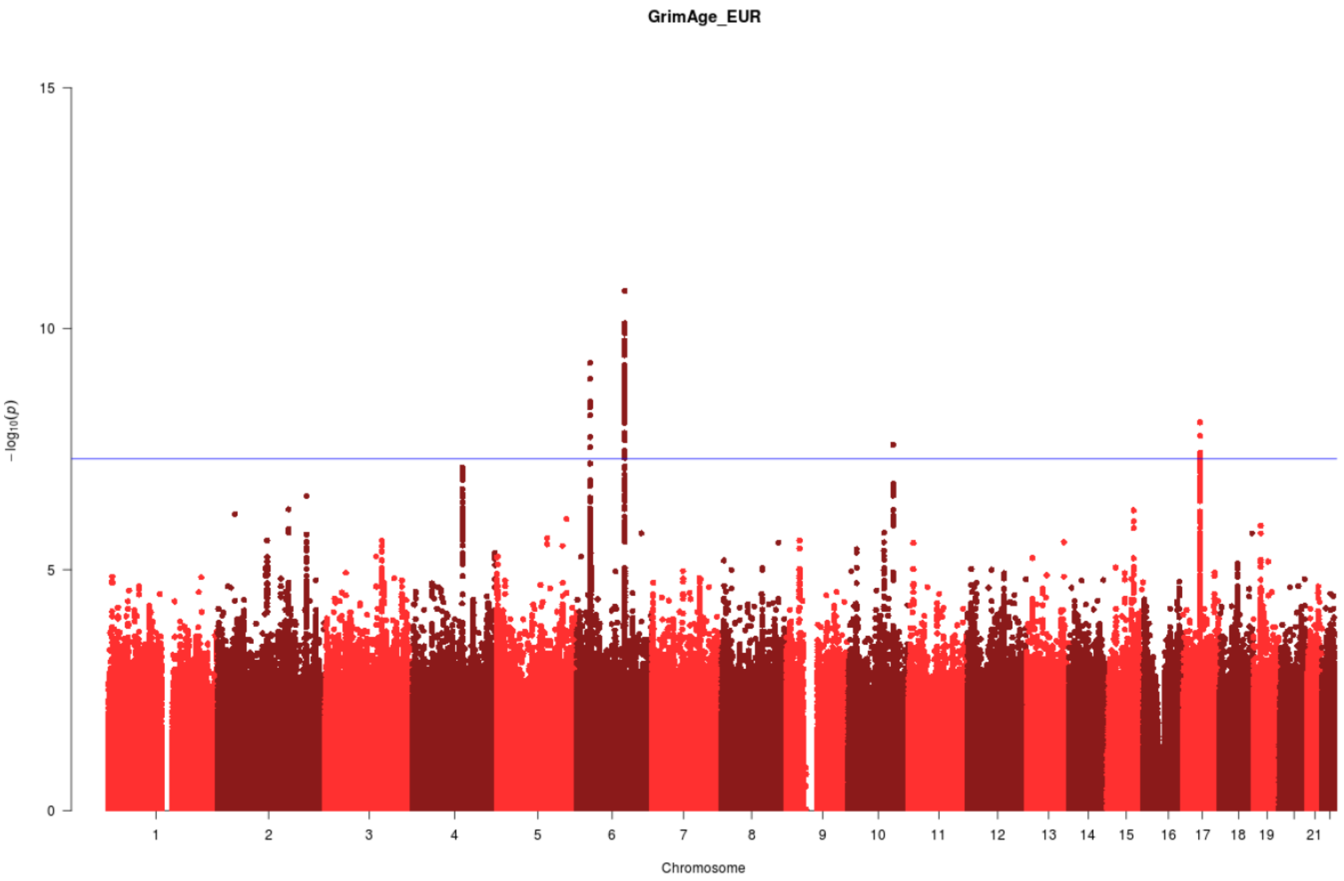

Fig S15: Manhattan Plot for GrimAge Acceleration in the European ancestry GWAS meta-analysis.

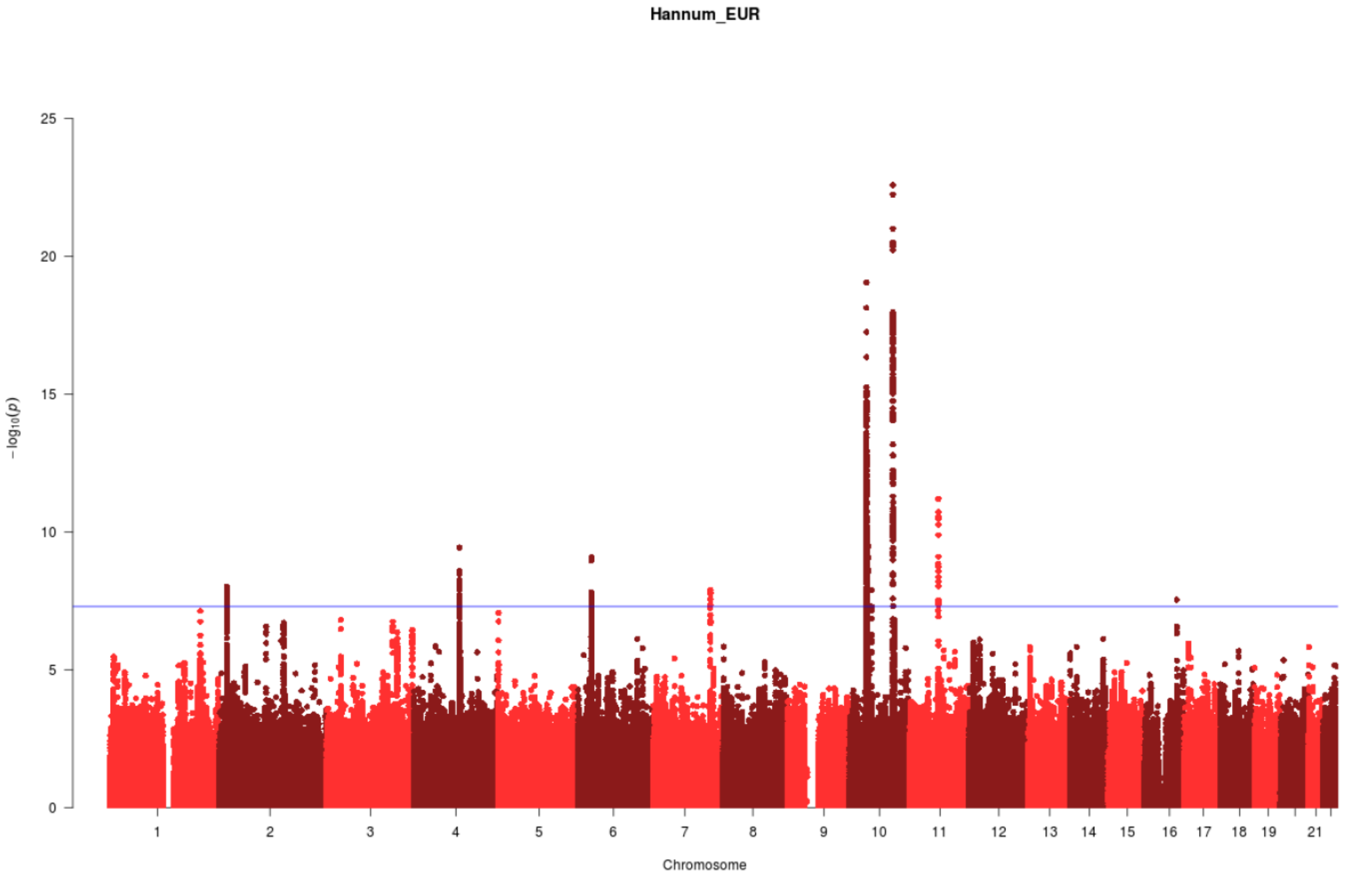

Fig S16: Manhattan Plot for Hannum Age Acceleration in the European ancestry GWAS meta-analysis.

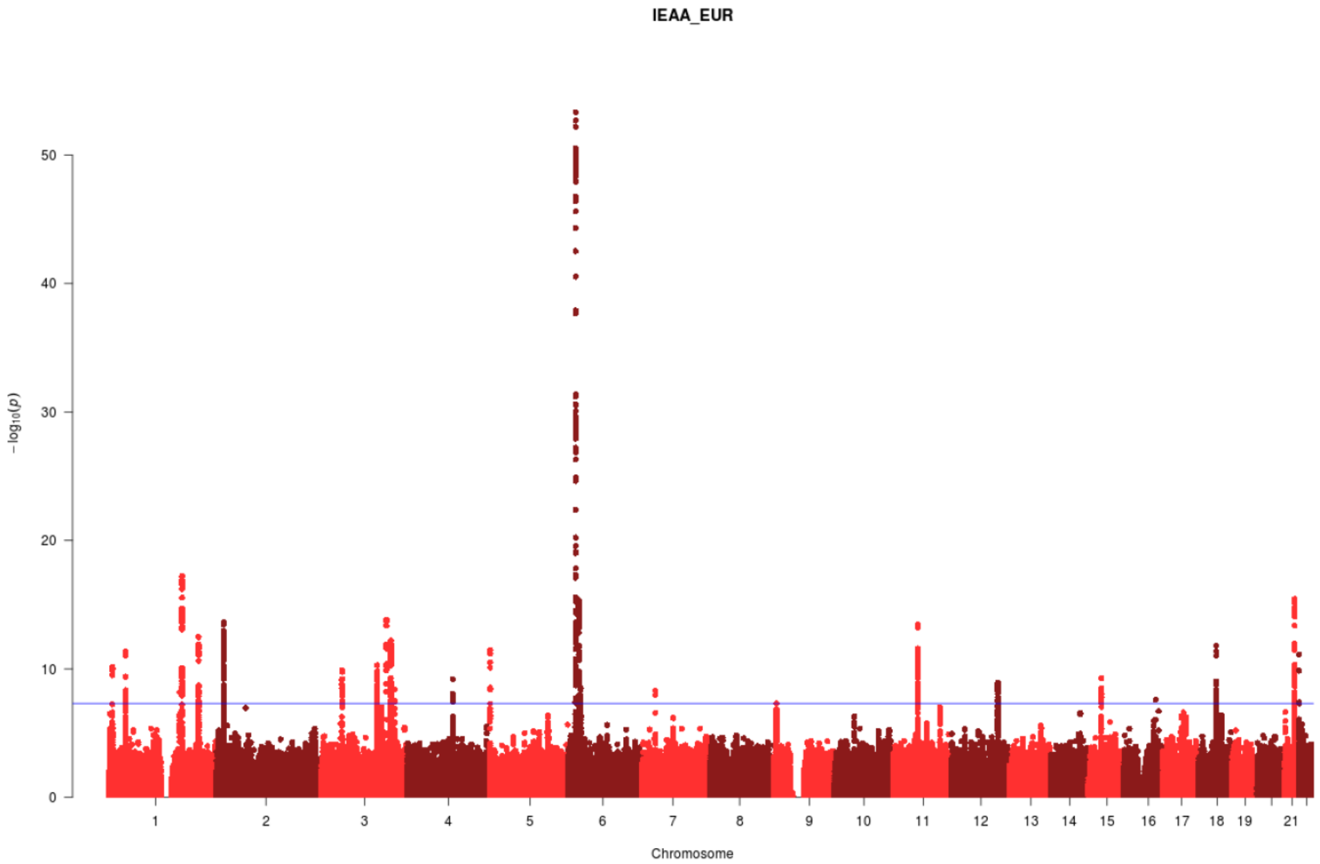

Fig S17: Manhattan Plot for IEAA in the European ancestry GWAS meta-analysis.

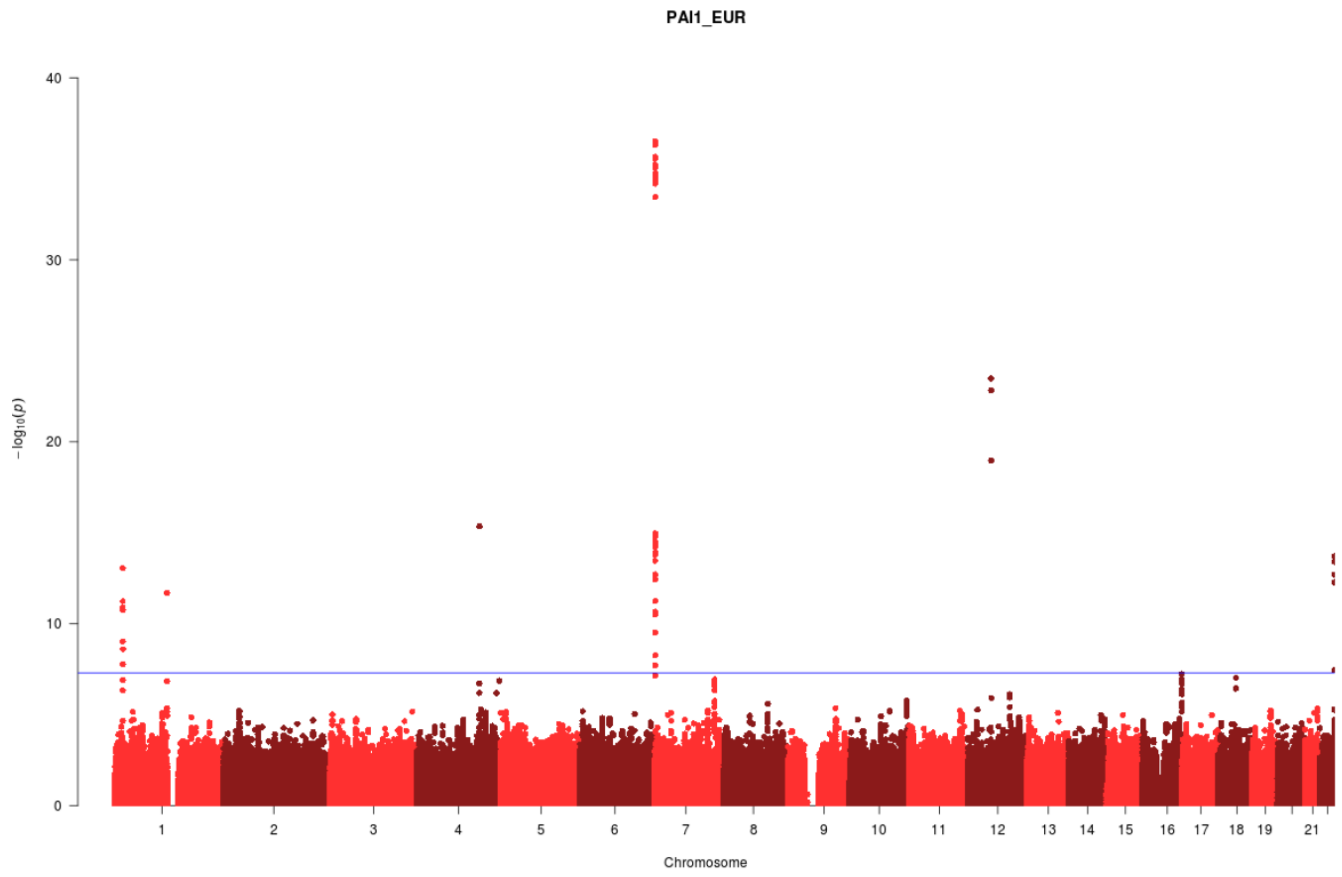

Fig S18: Manhattan Plot for DNAm PAI1 levels in the European ancestry GWAS meta-analysis.

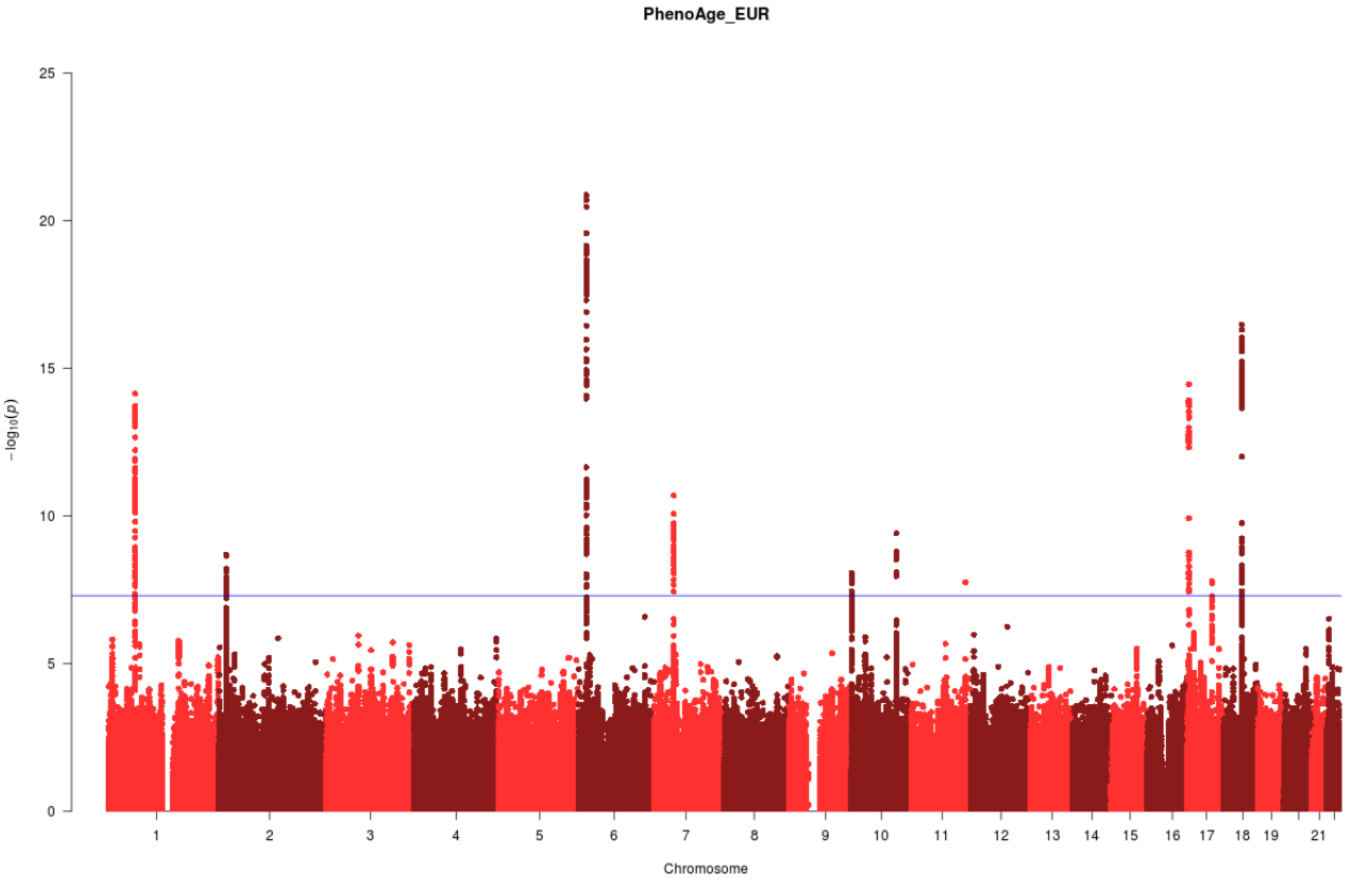

Fig S19: Manhattan Plot for PhenoAge Acceleration in the European ancestry GWAS meta-analysis.

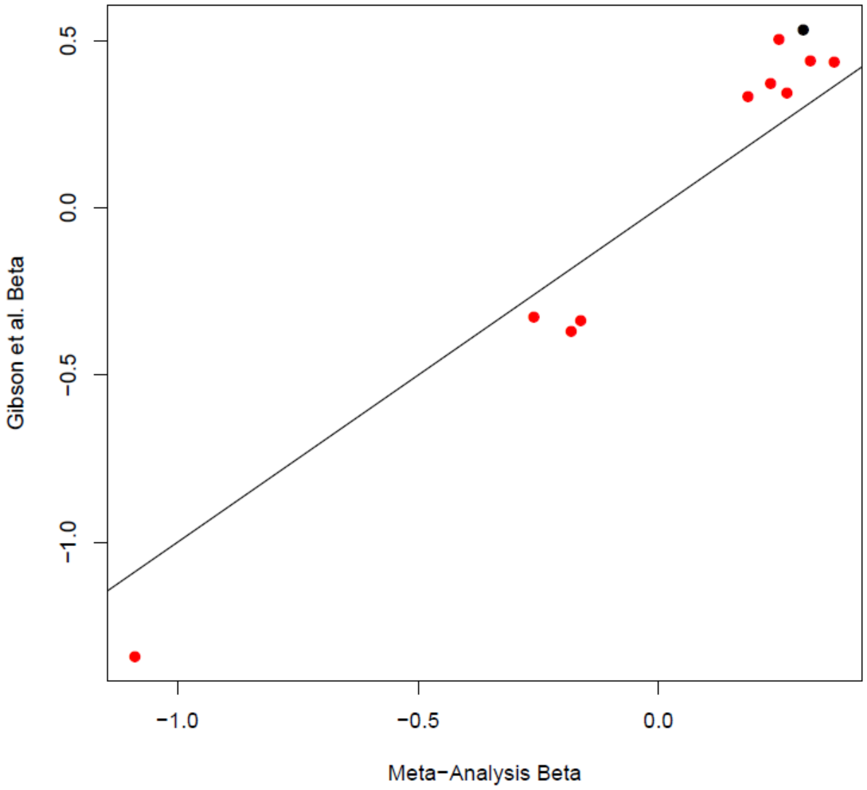

Fig S20: Plot of effect sizes for genome-wide significant SNPs in Gibson et al. vs effect sizes in a lookup of the current meta-analysis results for Hannum Age Acceleration and IEAA.

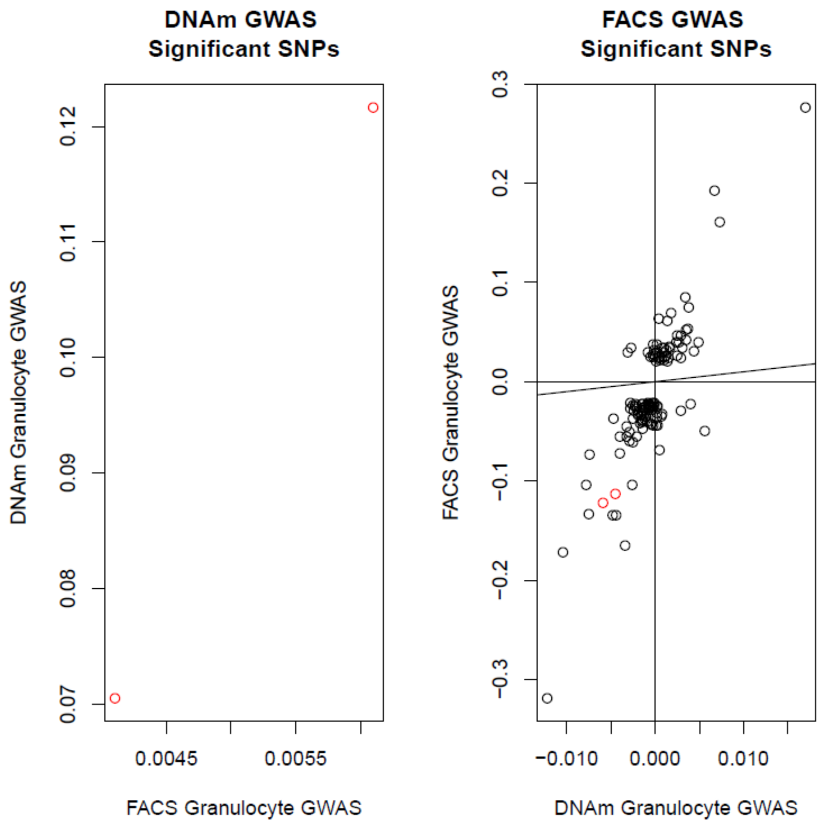

Fig S21: Plot of effect sizes for genome-wide significant SNPs in Astle et al. vs effect sizes in a lookup of the current meta-analysis results for DNAm granulocyte proportions (and vice versa).

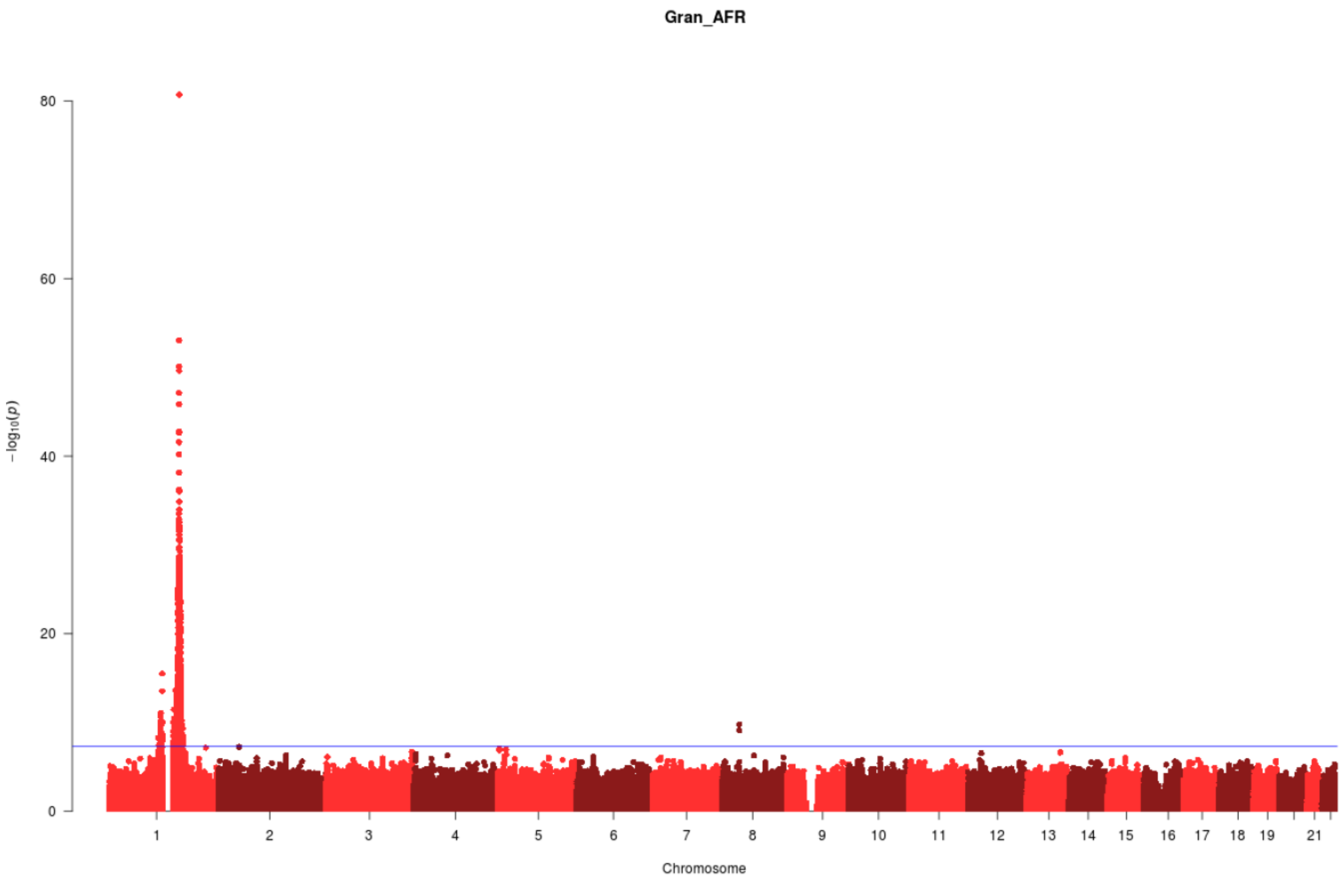

Fig S22: Manhattan Plot for DNAm granulocyte proportions in the African American GWAS meta-analysis.

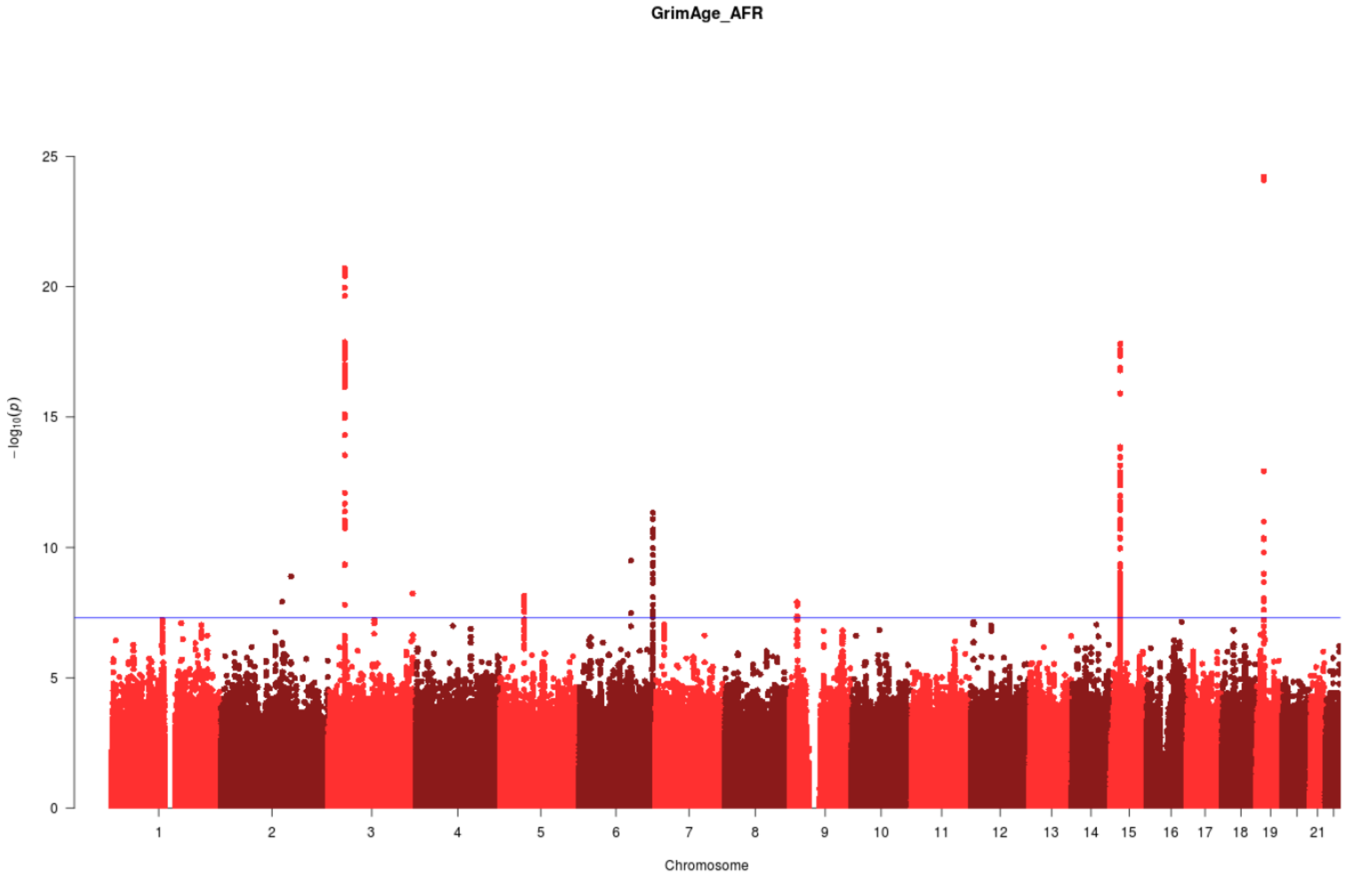

Fig S23: Manhattan Plot for GrimAge Acceleration in the African American GWAS meta-analysis.

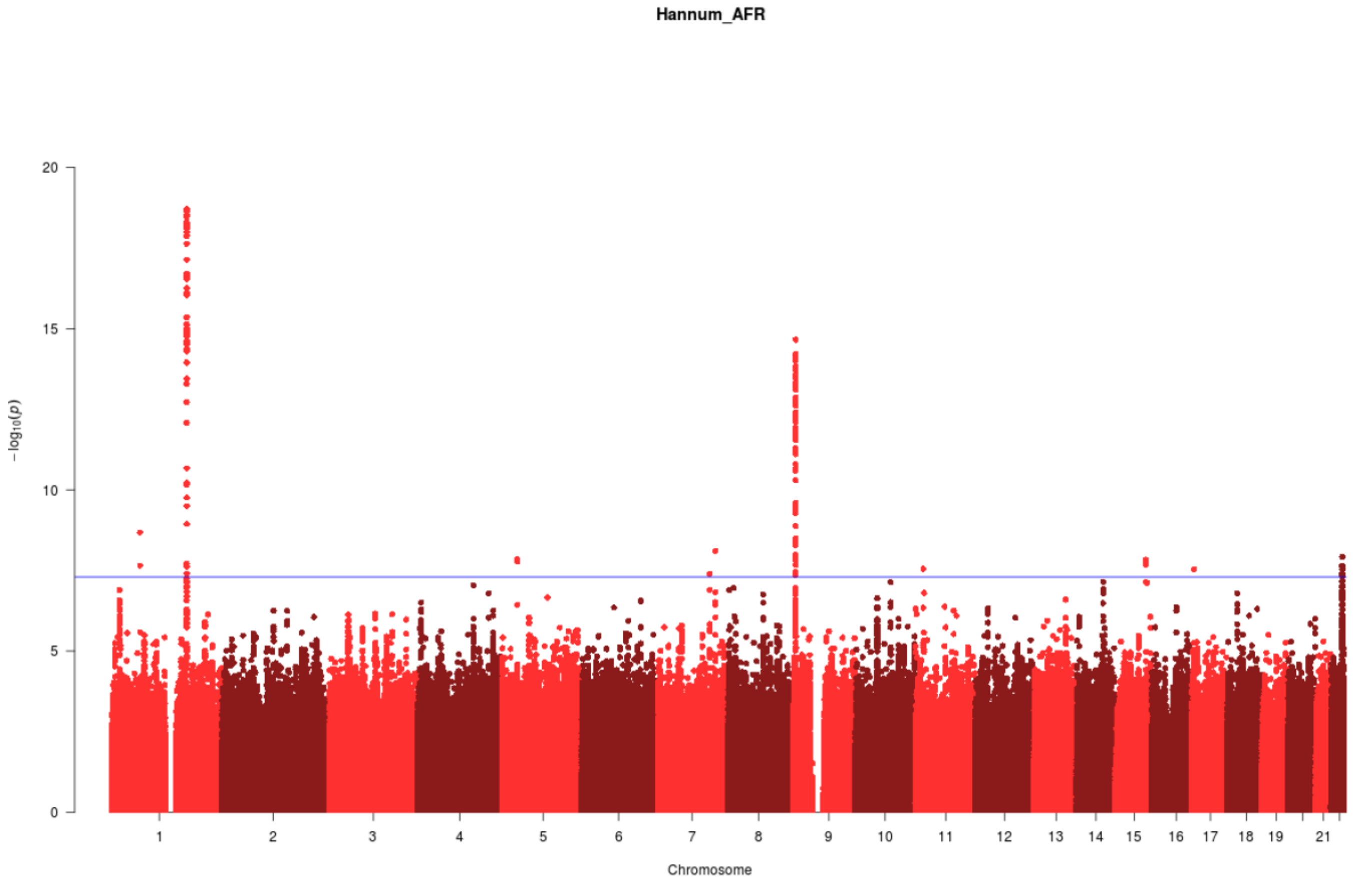

Fig S24: Manhattan Plot for Hannum Age Acceleration in the African American GWAS meta-analysis.

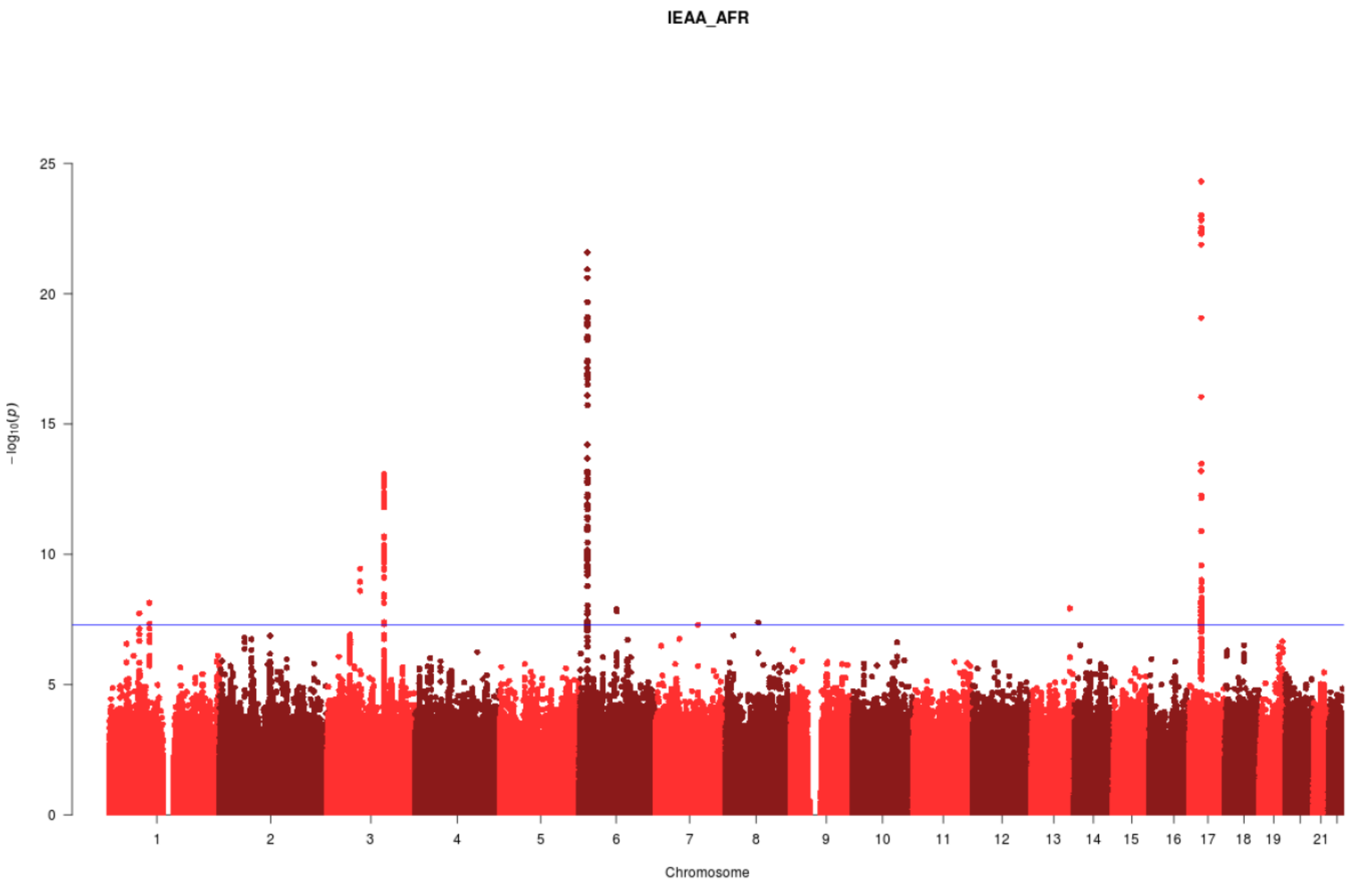

Fig S25: Manhattan Plot for IEAA in the African American GWAS meta-analysis.

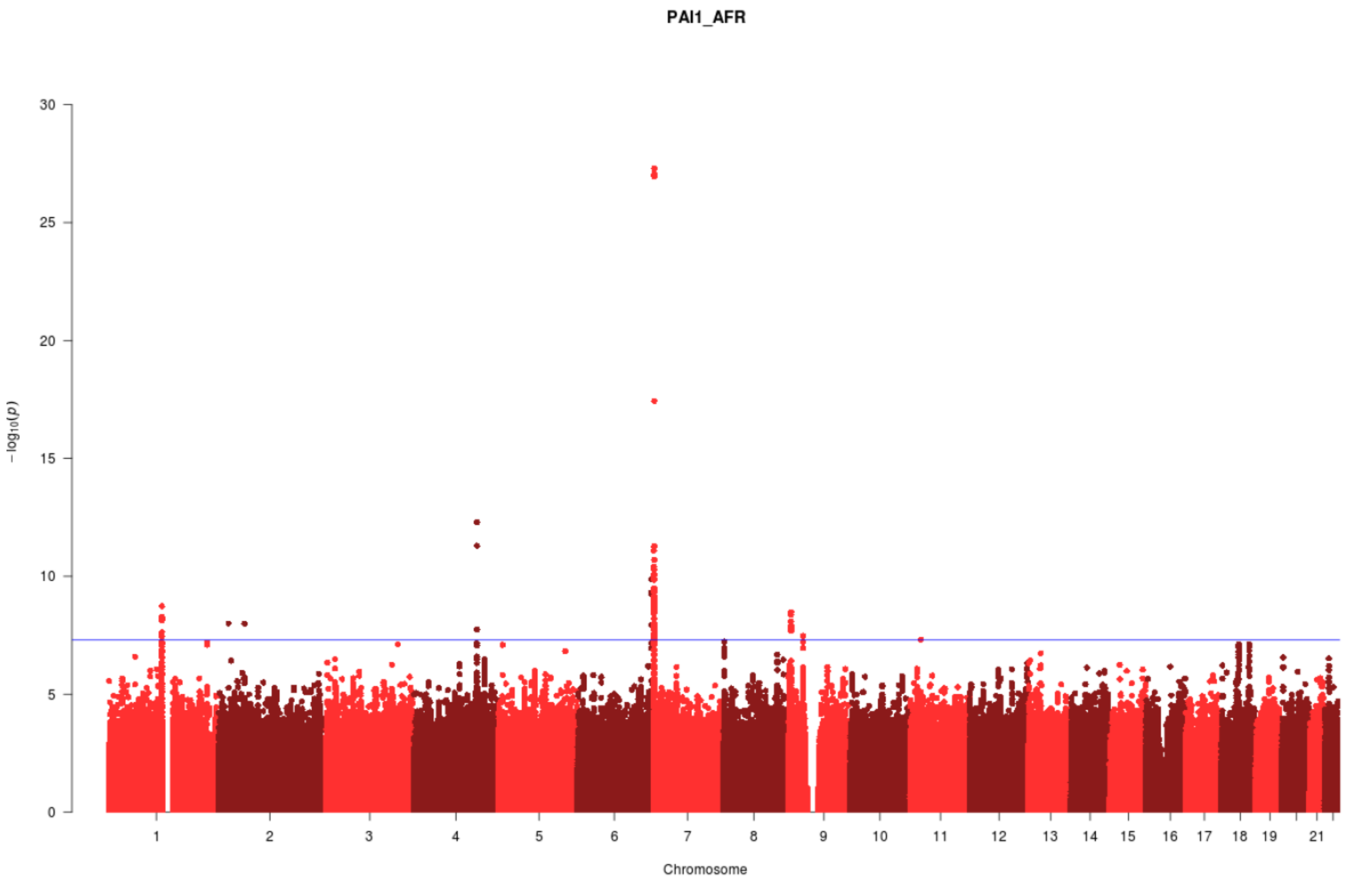

Fig S26: Manhattan Plot for DNAm PAI1 levels in the African American GWAS meta-analysis.

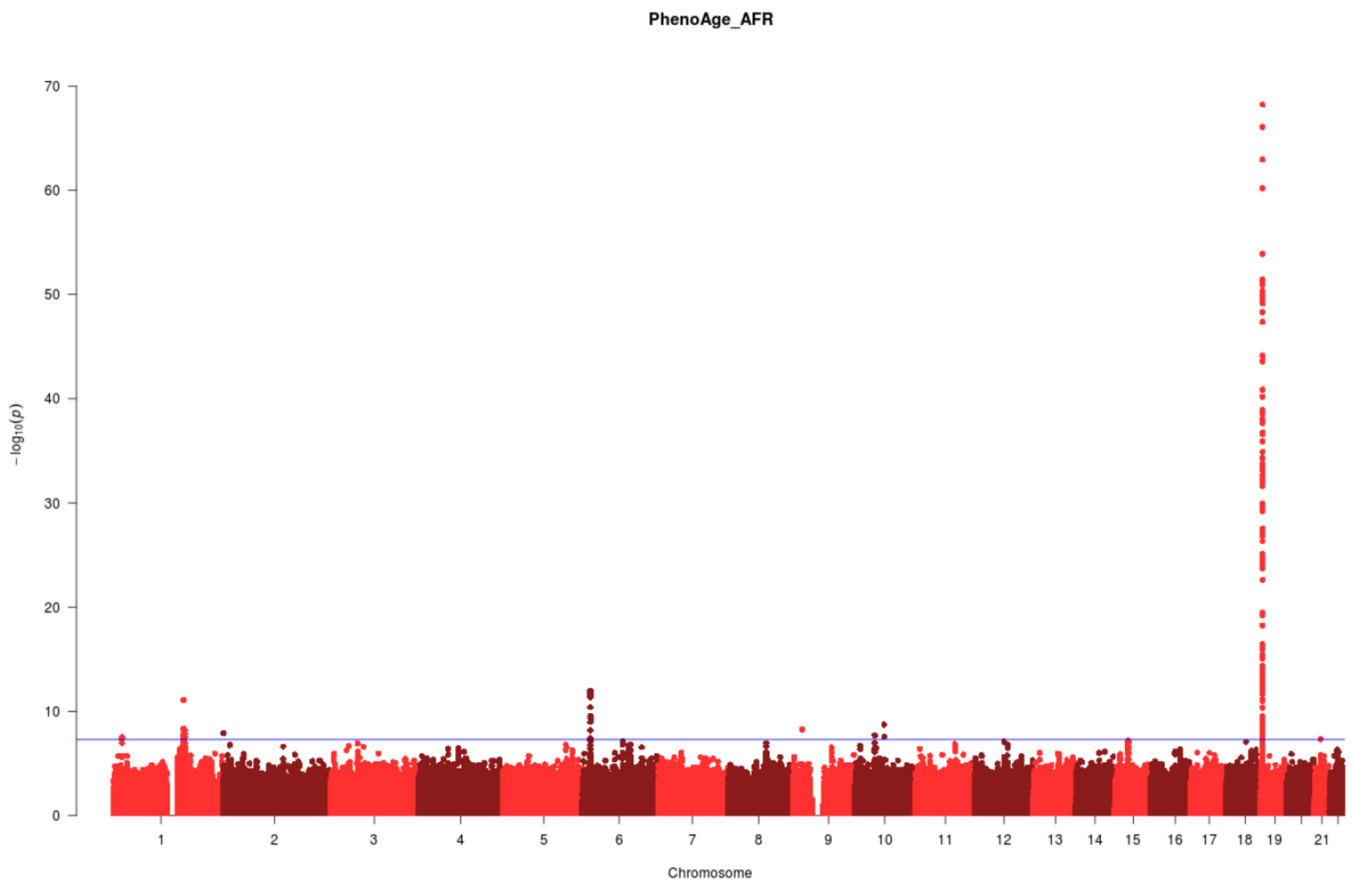

Fig S27: Manhattan Plot for PhenoAge Acceleration in the African American GWAS meta-analysis.

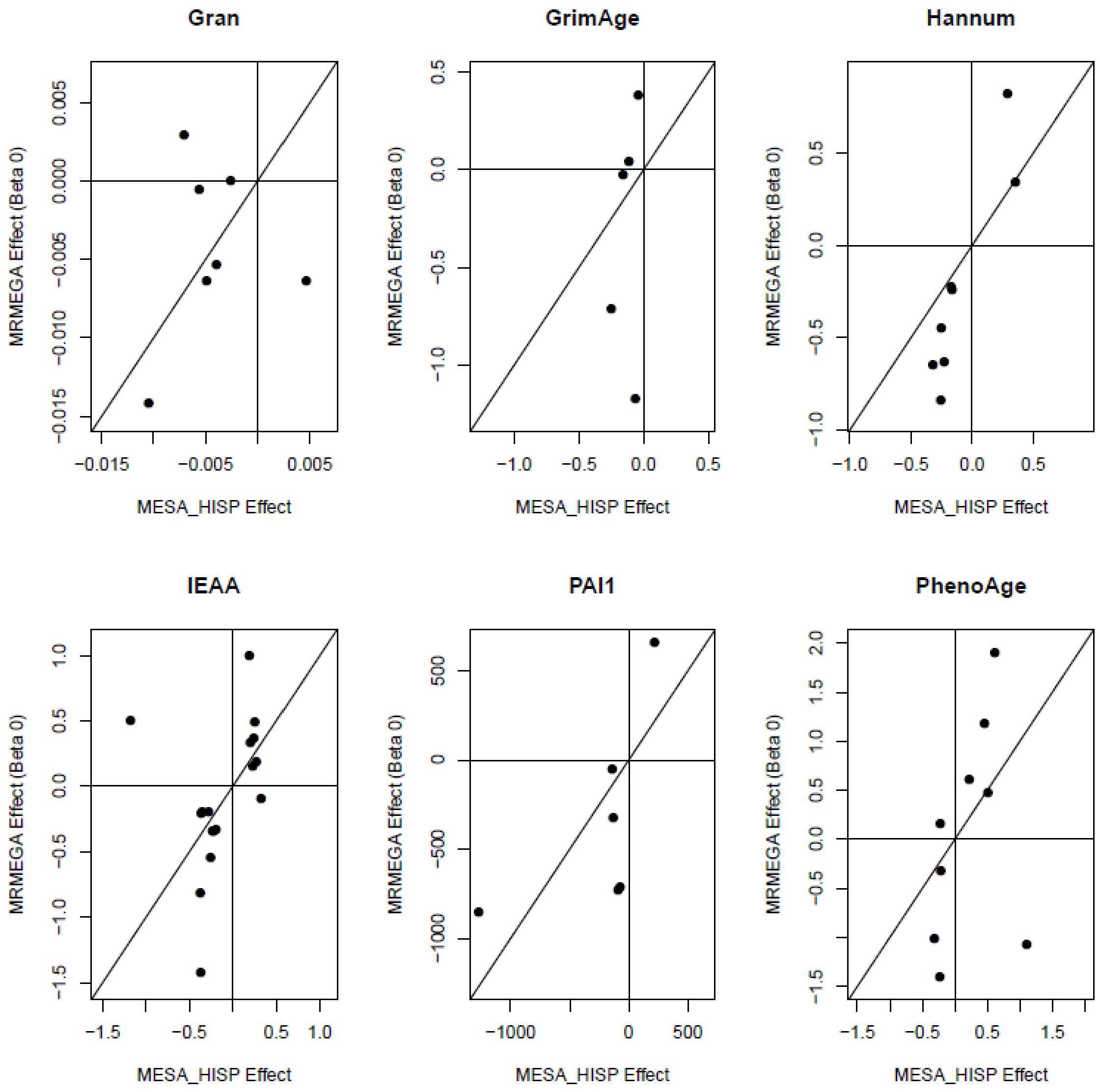

Fig S28: Plot of lead African American and European ancestry trans-ethnic meta-analysis SNP effect sizes against the same SNPs in the Hispanic subset of the MESA cohort.

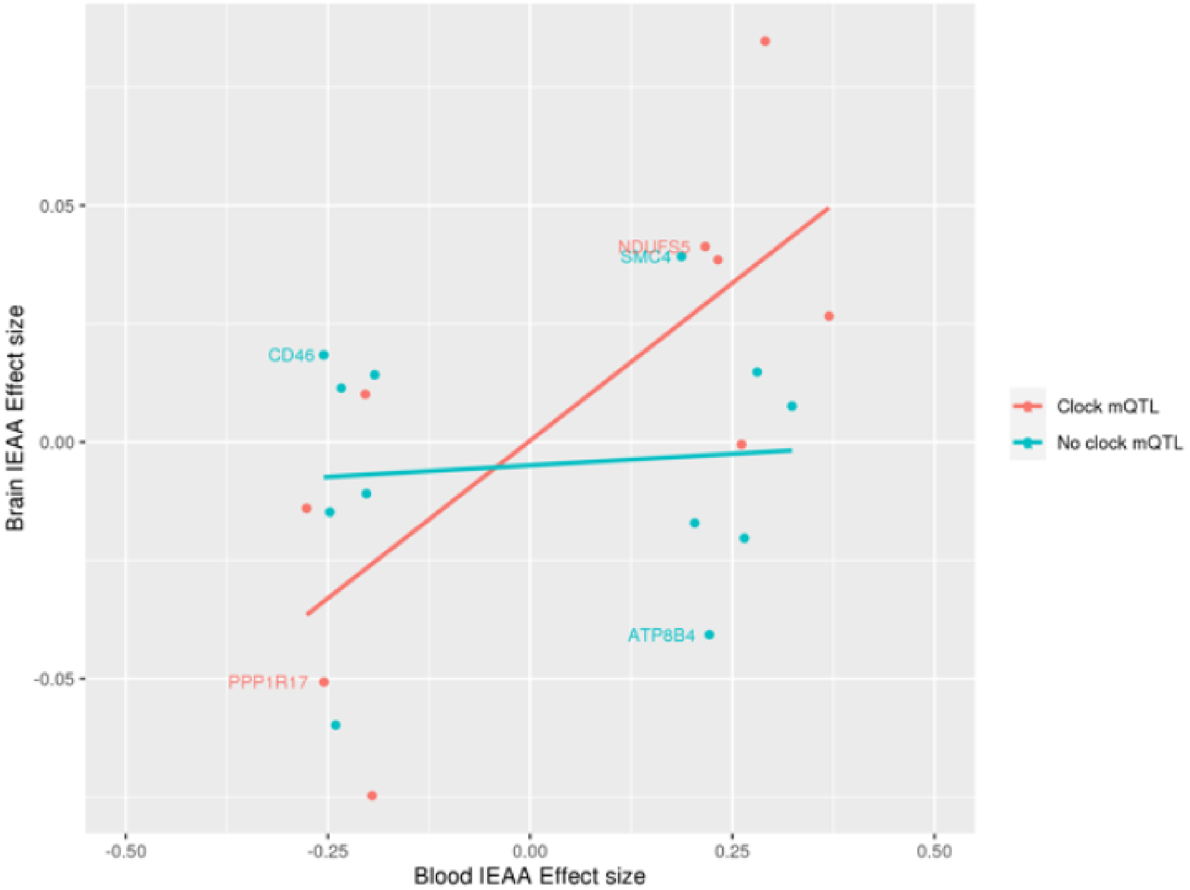

Fig S29: Lookup of 24 blood-based independent genome-wide significant SNPs for IEAA in a brain-based GWAS of IEAA (overlap of 21 SNPs). The red line represents the linear regression line (r=0.74) for SNPs that are also mQTLs for IEAA clock CpG sites. The turquoise line represents the linear regression line (r=0.08) for SNPs that are not mQTLs for IEAA clock CpGs. Labelled points correspond to loci where there was strong evidence of SNPs sharing genetic effects with eQTLs.

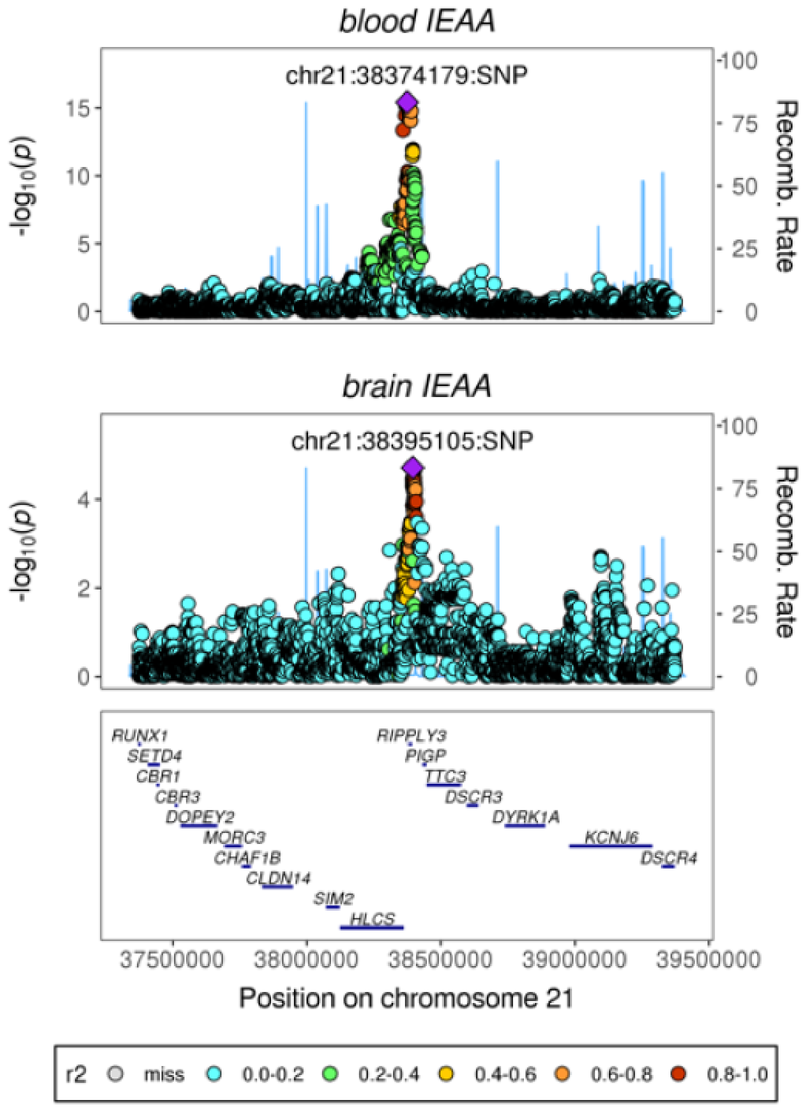

Fig S30: LocusZoom plot for the region (*DSRC6*/*RIPPLY3*) with highest evidence of genetic colocalization for the blood- and brain-based GWASs. Note that the lead SNP from the blood-based GWAS also colocalizes with a mQTL for an IEAA clock CpG, cg13450409 (PP=0.99, **Additional File 2: Table S11**).

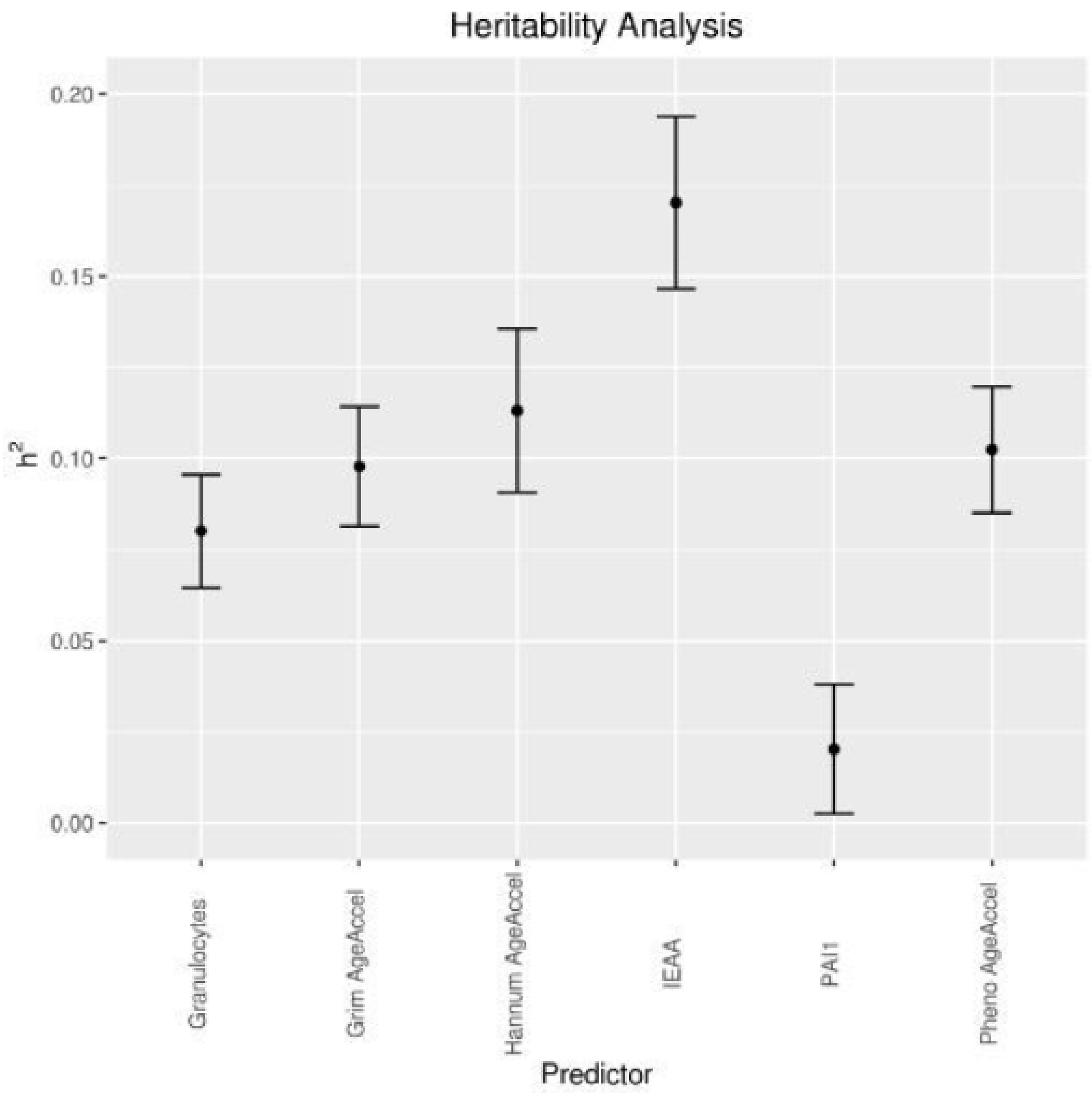

Fig S31: LD regression SNP-based heritability estimates for the six epigenetic biomarkers.
