# Supplementary figures and images for: Genome-wide association studies identify 137 genetic loci for DNA methylation biomarkers of aging
Source: Genome Biol. 2021 Jun 29;22:194. doi: 10.1186/s13059-021-02398-9 (PMC8243879; doi:10.1186/s13059-021-02398-9)

**Additional File 5:** Colocalization plots for 12 GWAS locus-transcript pairs reported in Table S15.

**
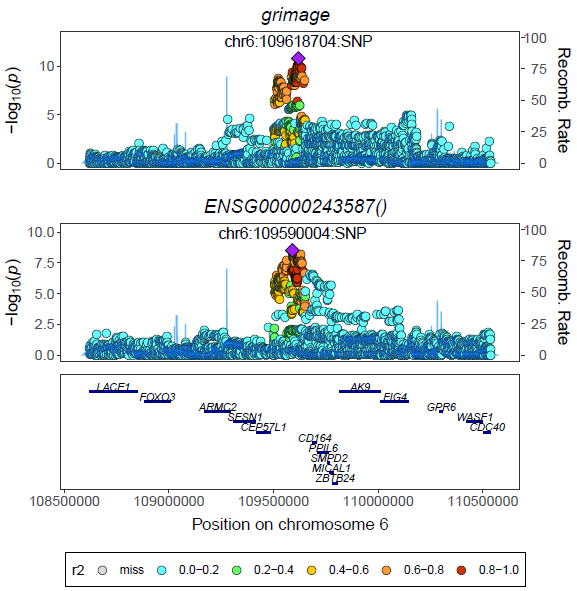
**


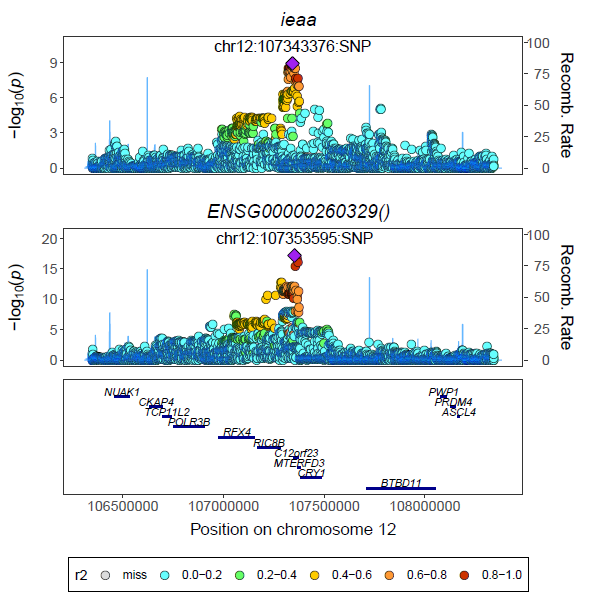


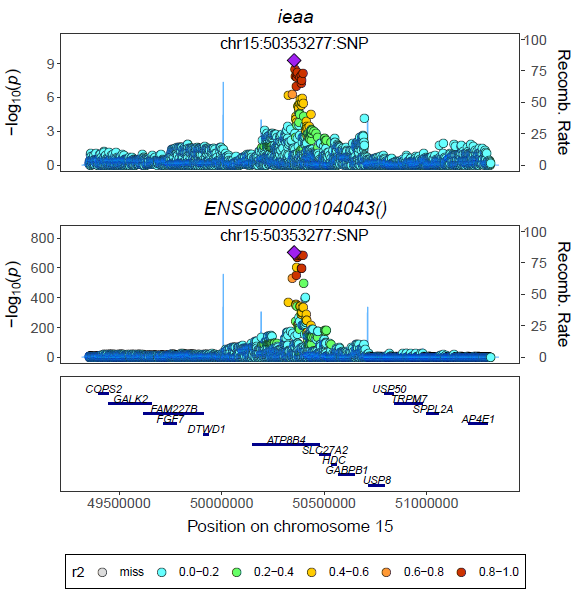


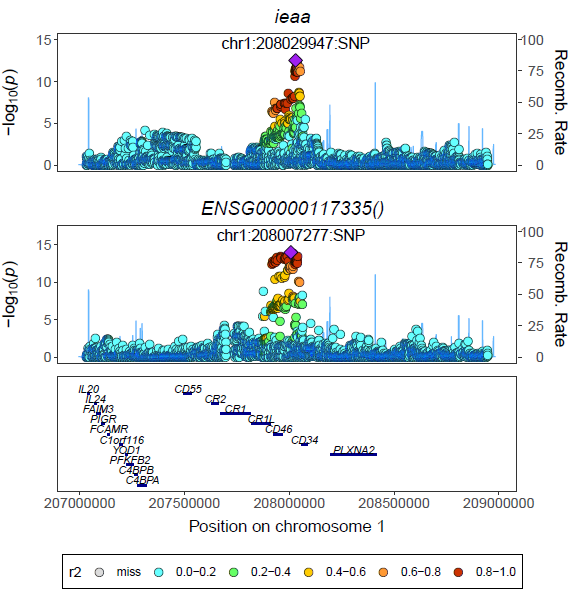


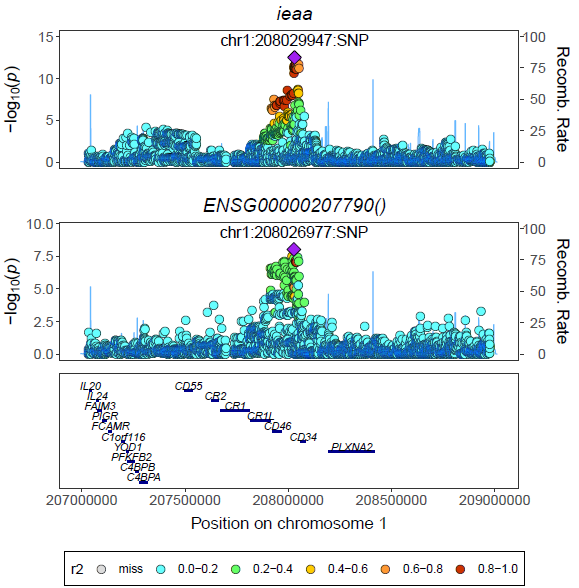


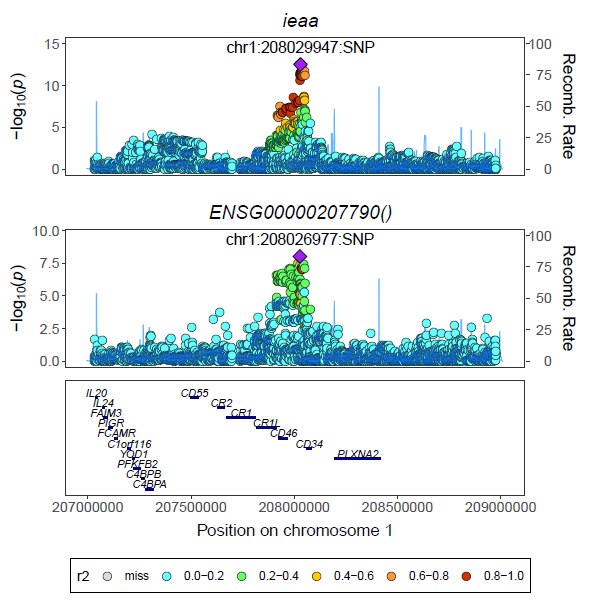


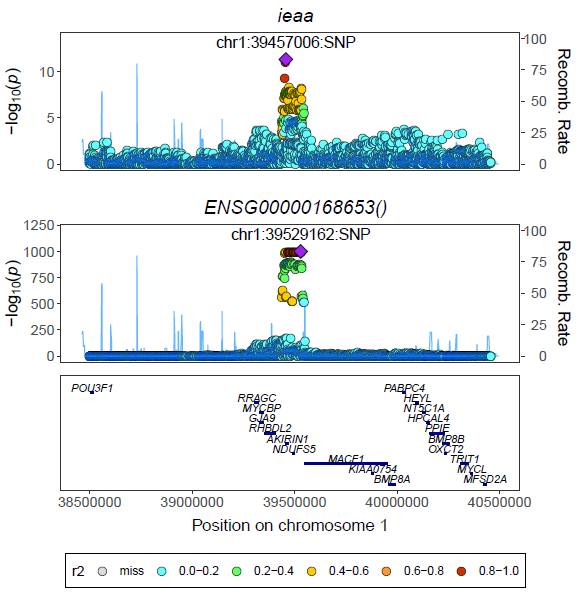


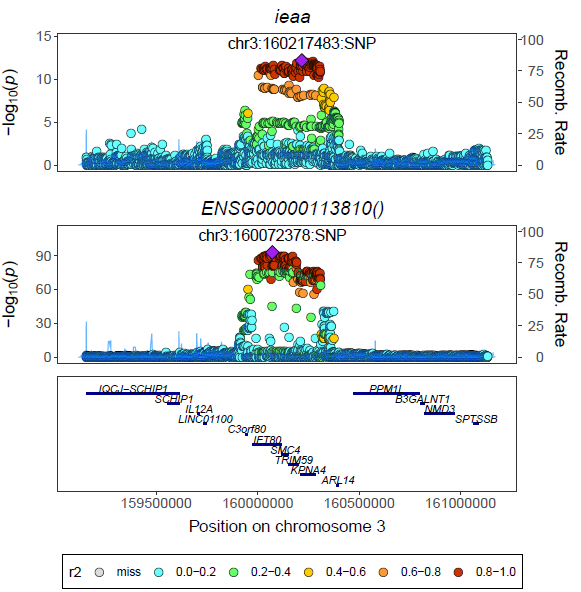


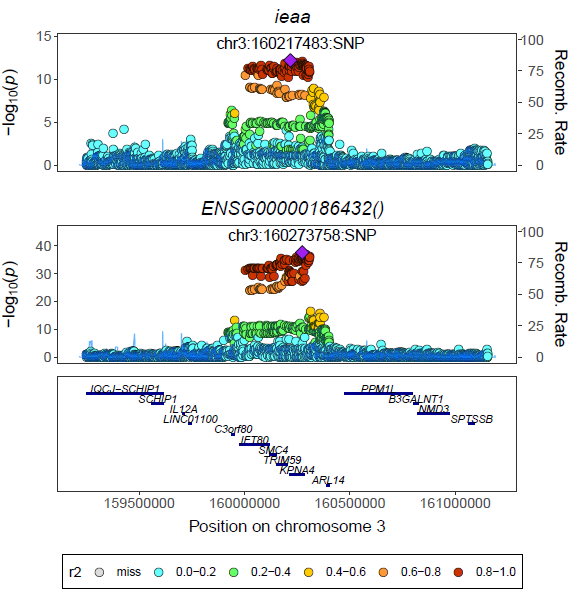


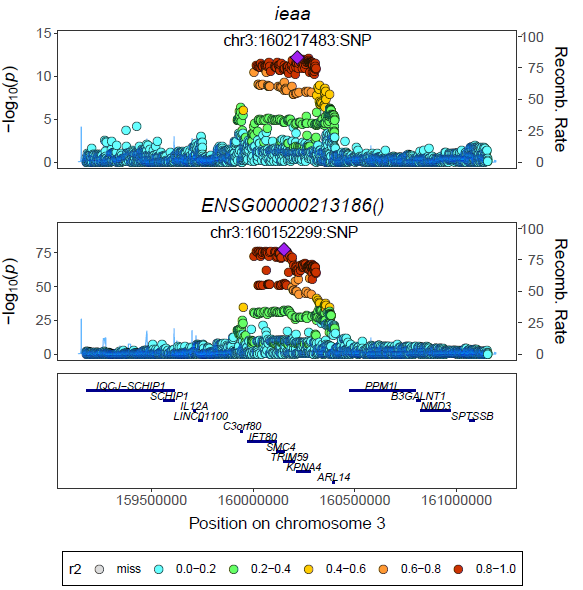


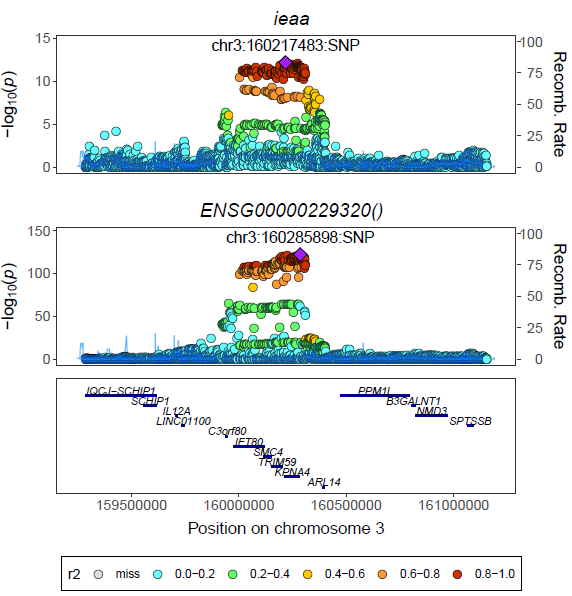


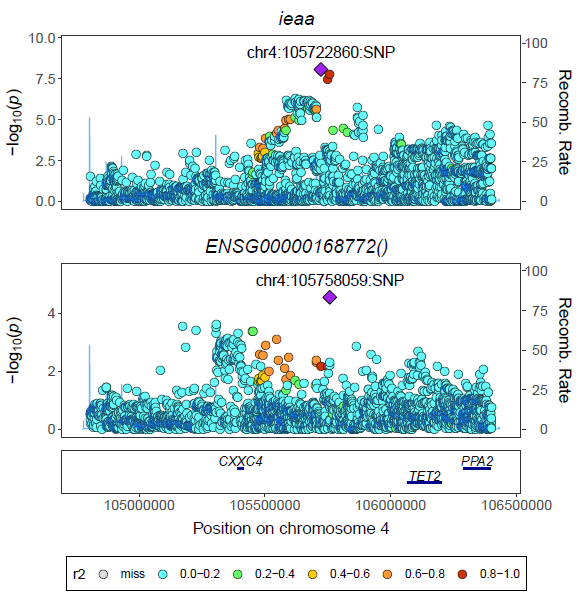

Supplement: Supplementary file 5 — Additional file 5. Colocalization plots. [file 13059_2021_2398_MOESM5_ESM.docx]
