# Supplementary material for: Genome-wide association studies identify 137 genetic loci for DNA methylation biomarkers of aging
Source: Genome Biol. 2021 Jun 29;22:194. doi: 10.1186/s13059-021-02398-9 (PMC8243879; doi:10.1186/s13059-021-02398-9)
Supplement: Supplementary file 6 — Additional file 6. Review history. [file 13059_2021_2398_MOESM6_ESM.docx]

**1^st^ round**

**Reviewer 1**

This manuscript focusses on a large meta-analysis to identify genetic variation that influences epigenetic clocks. I am afraid that I am not in a position to assess the statistics. However, the size of the study is indeed impressive and provides a better understanding of the genetic factors that can influence the various epigenetic clocks. This is likely to be of interest to a large group of researchers.

**Reviewer 2**

This GWAS of DNAm ageing clocks/biomarkers by McCartney et al. is massive step forward from previous efforts to identify genetic mechanisms of these ageing clocks. First, the authors identify many "novel" loci for several epigenetic clocks (novel in that they were not previously associated with DNAm) but crucially for me they test whether the clocks (which are very predictive of age, ageing outcomes, and mortality) are causally related to these outcomes, or are themselves biomarkers. The paper concludes the latter, and that epigenetic ageing/clocks are likely reflecting of other pathways/mechanisms that impact ageing, morbidity and ultimately longevity.

This is an excellent paper, with several major strengths. The use of several different ageing clocks (not just the Horvath epigenetic clock, but also PhenoAge which is completely different in its derivation) means the results are not limited to one or other method/training dataset, and the conclusions are more generally applicable to ageing "clocks". Of course, the sample size is a great improvement on previous efforts, and the inclusion of a significant sample of African-ancestry participants is very important for generalizing results away from European-only cohorts. The 69 loci in the trans-ethnic meta-analysis are therefore of particular interest. I enjoyed reviewing the paper, and though it is quite long I think all the extra/sub-analyses are necessary for such a complex set of traits.

I have one major comment. Studies of DNAm always set my hobby-horse rocking, and this is no exception: cell-type heterogeneity is an inescapable part of studying methylation (or indeed any epigenetic/gene expression data) clocks, as different proportions of the different cell types are known to be associated with age, morbidities, exposures, and DNAm. I realise the authors spent time in the methods detailing steps taken to minimize this, but given that one of the two gene-sets in the Mendelian disease genes section was "disorders of platelet function" this raised a flag in my mind that perhaps there is still unaccounted for cell-type effects here. In the Discussion the authors say "GWAS results that are shared between IEAA (which adjusts for differences in white blood cell proportions) and the other epigenetic clocks (Hannum clock, PhenoAge, GrimAge) are less likely to be influenced by differences in cell composition" but I think much more is needed, personally. First, only broad categories of white cell proportions are adjusted for, not the hundreds of sub-types that actually exist, and second, what is the evidence that the other epigenetic clocks are less influenced by different in cell composition? If a reference or rationale was provided here, I missed it. I don't think this undermines all the results of the study by any means, but more discussion is warranted, particularly how this could affect the MR analysis (surely this impacts some of the pleiotropy assumptions).

Overall though this is a superb study and very well-written and thorough paper. I look forward to seeing it in print.

**Authors’ response**

**Reviewer #1:** This manuscript focusses on a large meta-analysis to identify genetic variation that influences epigenetic clocks. I am afraid that I am not in a position to assess the statistics. However, the size of the study is indeed impressive and provides a better understanding of the genetic factors that can influence the various epigenetic clocks. This is likely to be of interest to a large group of researchers.

Response: We thank the reviewer for their positive comments

**Reviewer #2:** This GWAS of DNAm ageing clocks/biomarkers by McCartney et al. is massive step forward from previous efforts to identify genetic mechanisms of these ageing clocks. First, the authors identify many "novel" loci for several epigenetic clocks (novel in that they were not previously associated with DNAm) but crucially for me they test whether the clocks (which are very predictive of age, ageing outcomes, and mortality) are causally related to these outcomes, or are themselves biomarkers. The paper concludes the latter, and that epigenetic ageing/clocks are likely reflecting of other pathways/mechanisms that impact ageing, morbidity and ultimately longevity.

This is an excellent paper, with several major strengths. The use of several different ageing clocks (not just the Horvath epigenetic clock, but also PhenoAge which is completely different in its derivation) means the results are not limited to one or other method/training dataset, and the conclusions are more generally applicable to ageing "clocks". Of course, the sample size is a great improvement on previous efforts, and the inclusion of a significant sample of African-ancestry participants is very important for generalizing results away from European-only cohorts. The 69 loci in the trans-ethnic meta-analysis are therefore of particular interest. I enjoyed reviewing the paper, and though it is quite long I think all the extra/sub-analyses are necessary for such a complex set of traits.

Response: Thank you for these very kind comments. We are delighted that you enjoyed reviewing the paper!

I have one major comment. Studies of DNAm always set my hobby-horse rocking, and this is no exception: cell-type heterogeneity is an inescapable part of studying methylation (or indeed any epigenetic/gene expression data) clocks, as different proportions of the different cell types are known to be associated with age, morbidities, exposures, and DNAm. I realise the authors spent time in the methods detailing steps taken to minimize this, but given that one of the two gene-sets in the Mendelian disease genes section was "disorders of platelet function" this raised a flag in my mind that perhaps there is still unaccounted for cell-type effects here. In the Discussion the authors say "GWAS results that are shared between IEAA (which adjusts for differences in white blood cell proportions) and the other epigenetic clocks (Hannum clock, PhenoAge, GrimAge) are less likely to be influenced by differences in cell composition" but I think much more is needed, personally. First, only broad categories of white cell proportions are adjusted for, not the hundreds of sub-types that actually exist, and second, what is the evidence that the other epigenetic clocks are less influenced by different in cell composition? If a reference or rationale was provided here, I missed it. I don't think this undermines all the results of the study by any means, but more discussion is warranted, particularly how this could affect the MR analysis (surely this impacts some of the pleiotropy assumptions).

Response: We thank the reviewer for their thoughtful comments. Cell-type heterogeneity in DNAm studies is a hobby-horse that is well worth rocking! IEAA is a derivative of Horvath’s pan-tissue epigenetic clock, which, given its broad application, is likely to be less influenced by white cell heterogeneity than blood-based clocks. Furthermore, IEAA also regresses out DNAmbased estimates for naive CD8+ T cells, exhausted CD8+ T cells, plasmablasts, CD4+ T cells, natural killer cells, monocytes, and granulocytes. However, we acknowledge that other subtypes may still influence the findings.

While it is interesting that we observed enrichment of Mendelian disease genes for IEAA relating to platelet disorders, our MR analyses did not support evidence for a causal link between platelets and IEAA.

We have added the following text (new text in bold) to the discussion to highlight these points:

“**The IEAA clock is a derivative of Horvath’s pan-tissue predictor that regresses out DNA methylation-based estimates for naive CD8+ T cells, exhausted CD8+ T cells, plasmablasts, CD4+ T cells, natural killer cells, monocytes, and granulocytes. Although other cell sub-types may influence the findings, the** GWAS results that are shared between IEAA (~~which adjusts for differences in white blood cell proportions~~) and the other epigenetic clocks (Hannum clock, PhenoAge, GrimAge) are less likely to be influenced by differences in cell composition**. However, there was still an enrichment in the GWAS results for IEAA and GrimAge with findings from previous white blood cell GWASs, such as basophils. Mendelian randomisation analyses indicated putative causal effects of lymphocyte count on PhenoAge, GrimAge, and Hannum age acceleration but not on IEAA. Furthermore, whereas we observed enrichment of Mendelian disease genes for IEAA relating to platelet disorders, the Mendelian randomisation analyses did not support evidence for a causal link between platelets and IEAA**.”

The reviewer also makes an excellent point regarding the MR analyses. We have added the following text (new text in bold) to the discussion to reference this:

“Future multivariate MR analyses will be required to test whether the protective causal effect of education on GrimAge is mediated by smoking, obesity, or other factors. **These studies could also explore potential pleiotropy between the clocks, health outcomes and white blood cell proportions**.”

Overall though this is a superb study and very well-written and thorough paper. I look

forward to seeing it in print.

Response: Thank you so much!

.
